# Identification and comparative analysis of the microRNA transcriptome in roots of two contrasting tobacco genotypes in response to cadmium stress

**DOI:** 10.1038/srep32805

**Published:** 2016-09-26

**Authors:** Xiaoyan He, Weite Zheng, Fangbin Cao, Feibo Wu

**Affiliations:** 1 Department of Agronomy, College of Agriculture and Biotechnology, Zijingang Campus, Zhejiang University, Hangzhou 310058, P.R. China

## Abstract

Tobacco (*Nicotiana tabacum* L.) is more acclimated to cadmium (Cd) uptake and preferentially enriches Cd in leaves than other crops. MicroRNAs (miRNAs) play crucial roles in regulating expression of various stress response genes in plants. However, genome-wide expression of miRNAs and their target genes in response to Cd stress in tobacco are still unknown. Here, miRNA high-throughput sequencing technology was performed using two contrasting tobacco genotypes Guiyan 1 and Yunyan 2 of Cd-sensitive and tolerance. Comprehensive analysis of miRNA expression profiles in control and Cd treated plants identified 72 known (27 families) and 14 novel differentially expressed miRNAs in the two genotypes. Among them, 28 known (14 families) and 5 novel miRNAs were considered as Cd tolerance associated miRNAs, which mainly involved in cell growth, ion homeostasis, stress defense, antioxidant and hormone signaling. Finally, a hypothetical model of Cd tolerance mechanism in Yunyan 2 was presented. Our findings suggest that some miRNAs and their target genes and pathways may play critical roles in Cd tolerance.

Cadmium (Cd), widely recognized as a dangerous environmental pollutant, ranks the first among the top six toxic metals (Cd, Cr, Cu, Hg, Ni and Pb) released into ecosystems^1^. When accumulated in plants, it not only causes various pathological symptoms, such as chlorosis, wilting, oxidative stress and cell death, but also affects crop yield and brings risks to human health *via* contamination of the food chain^2^. Thus, it is of great importance to understand mechanisms of Cd accumulation and tolerance for minimizing Cd accumulation in plants. Previous studies have shown that Cd-related genes such as transporters are responsible for Cd uptake and sequestration^3^. Recent studies have demonstrated that heavy metal-related gene expression can be regulated by a group of microRNAs (miRNAs)^4^,^5^,^6^.

miRNAs, being a class of short, endogenous, noncoding small RNAs that base-pair with their target mRNAs to induce silencing of their target genes have become a major focus of research in molecular biology^7^. A growing number of miRNAs have been isolated and characterized from several plant species^8^,^9^. They have been demonstrated to be involved in many biological and metabolic processes, including developmental regulation, growth control, cell differentiation, signal transduction, and biotic and abiotic stresses^10^. Analysis of miRNAs and their targets involved in heavy metal stress mediation will give new knowledge towards a better understanding of plant stress response mechanisms. To achieve this goal, high-throughput sequencing technology has become a powerful tool for discerning the trascriptome of different tissues. For example, using this approach, 84 miRNAs (including 19 new miRNAs of 18–24 nucleotides) have been identified from *Brassica napus* seedlings exposed to Cd, most being differentially regulated by this heavy metal^5^.

Tobacco, which is an important economic and agricultural crop in China as well as around the world, has recently been investigated as a potential biofuel crop. However, Cd contamination in the soil seriously affects tobacco yield and quality in many areas. Thus, there is an urgent need to improve Cd tolerance in tobacco. Guo *et al*.^11^ successfully identified 262 miRNAs from tobacco roots, before and after topping, using next-generation deep sequencing^11^. Gao *et al*.^12^ demonstrated that 165 conserved miRNAs and 50 novel miRNAs from tobacco leaves, stems and roots were responsible for tobacco development using high-throughput sequencing technology^12^. However, it is unclear whether these miRNAs are responsive to Cd tolerance and whether they are involved in any reduction of Cd content in tobacco. Furthermore, little is known about the relative abundance and the expression specificity of these miRNAs in tobaccos differing in Cd tolerance. Therefore, our aim was to determine if certain miRNAs have ability to withstand Cd stress in tobacco plants.

In the present study, genome-wide identification and analysis of miRNAs in roots of two tobacco genotypes differing in Cd tolerance was conducted using high-throughput sequencing technology. In addition, an integrated schematic diagram was proposed based on the identified Cd tolerance associated miRNAs to better understand the Cd-tolerant mechanism in tobacco. Meanwhile, a number of novel mRNAs were added to the tobacco microRnome. The results shed new light on the understanding of tobacco miRNAs which may play a role in Cd tolerance.

## Results

### Effects of Cd on plant growth and Cd content

After 5 d Cd exposure, SPAD value, plant height, root length, fresh weight and biomass were reduced by Cd treatment; however, Yunyan 2 was less affected than Guiyan 1 (Supplemental Table S1). To evaluate Cd tolerance, the following formula was derived to give an integrated score of tolerance for each cultivar: integrated score = [SPAD value* × 0.1429 + shoot height* × 0.1429 + root length* × 0.1429 + fresh weight* × 0.1429 + dry weight* × 0.1429] (*=percentage reduction or increase in growth/physiological parameters relative to the controls) (Supplemental Table S1). There is a negative correlation between Cd tolerance and the integrated scores; Yunyan 2 showed a greater Cd tolerance with a lower score than Guiyan 1. After 5 d Cd exposure, Cd concentrations in shoots and roots had significantly increased in Guiyan 1 and Yunyan 2 compared with the controls. The shoot/root Cd concentrations and Cd accumulation per plant were 6.8%/7.2% and 30.6% higher in Yunyan 2 than in Guiyan 1 under Cd stress (Supplemental Fig. S1).

### Deep-sequencing analysis of tobacco small RNAs

A total of approximately 27.2 million raw reads with lengths of 10 to 35 nt were generated from four root libraries (G − Cd, G + Cd, Y − Cd and Y + Cd), using Solexa high-throughput sequencing (Table 1). After filtration, a total of 6 561 741 (94.7%), 6 945 431 (93.2%), 5 790 967 (93.1) and 5 631 677 (85.9%) clean reads corresponding to 2 013 956 (29.0%), 1 394 044 (18.7%), 2 076 409 (33.4%) and 1 061 349 (16.2%) unique clean reads were obtained for the G − Cd, G + Cd, Y − Cd and Y + Cd libraries, respectively. As the tobacco genome is not fully sequenced, a BLAST search was implemented against tobacco ESTs and GSS database. When total reads were analyzed, 914 597–1 765 463 (14.0–28.4%) and 1 005 777–2 604 528 (15.3–41.9%) reads could be matched to the ESTs and GSS database corresponding to only 154 010–561 780 (2.4–9.0%) and 221 124–1 162 110 (3.4–18.7%) unique reads for the four root libraries. Furthermore, 9 059–25 408 (0.1–0.4%) and 24 683–179 792 (0.4–3.0%) specific reads in each library were matched to the ESTs and GSS database, respectively. In addition, a search of the miRBase database (v20.0) revealed 24 296 (0.4%), 33 746 (0.5%), 22 924 (0.4%) and 36 649 (0.6) unique matches for the small RNAs identified in G − Cd, G + Cd, Y − Cd and Y + Cd libraries, respectively.

**Figure 1:**
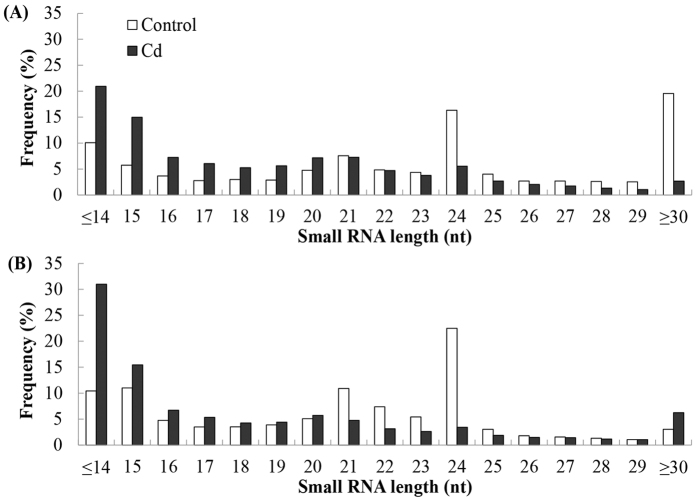
Length distribution of small RNAs in the roots of control (open bars) and Cd-treated (filled bars) Guiyan 1 (**A**) and Yunyan 2 (**B**) plants. Y-axis: the percentage of small RNA reads. G − Cd, G + Cd, Y − Cd and Y + Cd correspond to hydroponically grown tobacco of the cultivars Guiyan 1 and Yunyan 2 grown in basic nutrition solution (BNS) or BNS + 50 μM Cd.

Regarding the length distribution of the small RNAs, the 21 and 24 nt sequences were dominant in G − Cd and Y − Cd libraries with 24 nt being the most abundant length (Fig. 1). However, for the G + Cd and Y + Cd libraries, the dominant small RNA length was 15 nt. For G − Cd and Y − Cd libraries, the number of 21 nt and 24 nt small RNAs of Yunyan 2 were 27.2% and 21.6% higher than Guiyan 1, respectively. However, for G + Cd and Y + Cd libraries, there were 65.2% and 76.5% more 21 nt and 24 nt small RNAs in Guiyan 1 than in Yunyan 2. Although the small RNAs greater than 30 nt with a large number in G − Cd library, the number of small RNAs greater than 30 nt was decreased sharply in G + Cd library by 86.2% compared to the G − Cd library.

### Identification of conserved and novel miRNAs

The miRBase database (v20.0), which contains 7 384 miRNAs (4 025 unique miRNAs) belonging to 72 plant species from 1 892 miRNA families, was used in this study. A total of 117 611 unique small RNA sequences from four root small RNA libraries of the G − Cd, G + Cd, Y − Cd and Y + Cd were used as queries to search the potential miRNAs in tobacco. Only perfect or near-perfect matches (up to 2 mismatches) were used to identify conserved miRNAs in this research. Based on the sequence similarity, our analysis identified 164 known miRNAs belonging to 53 miRNA families (Supplemental Table S2). Of these 53 miRNA families, 3 families, miR169, miR156 and miR172, were identified containing 19, 10 and 10 miRNAs, respectively; 28 families contained two to eight miRNAs; and 22 families were only represented by a single miRNA (Supplemental Fig. S2). Among 164 identified known miRNAs, a length 21 nt was dominant, containing 101 miRNAs (Supplemental Fig. S3).

RNAfold and Mireap were used to identify novel tobacco miRNAs. A total of 30 small RNAs met the criteria and were considered putative, novel, tobacco miRNAs (Supplemental Table S3). These novel candidate miRNAs displayed a length distribution between 20 nt and 23 nt, with a peak at 22 nt containing 11 miRNAs (Supplemental Fig. S3). The adjusted minimum free energy (AMFE) varied from −65.1 to −25.2 kcal mol^−1^, which is similar to the free energy values of other plant miRNA precursors. The predicted hairpin structures for the precursors of these miRNAs required sequence lengths from 50 to 313 nt, and all of these 30 novel miRNAs have secondary structures with characteristic stem-loop hairpins (Supplemental Fig. S4).

### Cd responsive miRNAs

**Figure 2:**
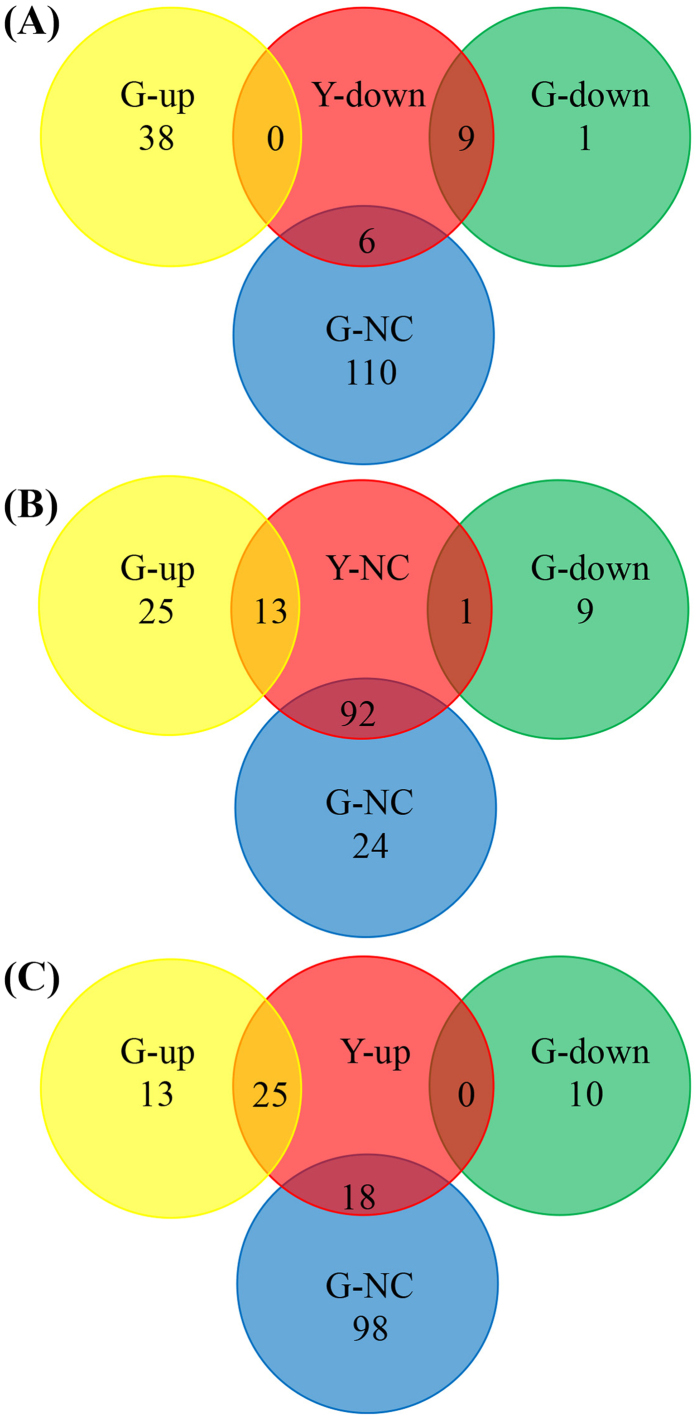
Root transcriptome profiles of Cd stress-responsive known miRNAs in tobacco. Venn diagrams show the number of miRNAs regulated by Cd treatment (50 μM Cd stress for 5 days) and overlap between the two genotypes: Guiyan 1 (G) and Yunyan 2 (Y). The data are overlaps of miRNAs in Guiyan 1, which were down-regulated (down) (**A**), no change (NC) (**B**) and up-regulated (up) (**C**) in Yunyan 2. Within each genotype, fold change (Cd vs control) is log_2_N, where changes in log_2_N ≥ 1.5 are considered as up-regulated, between 0 < |log_2_N| < 1.5 are considered as unchanged and log_2_N ≤ -1.5 are considered as down-regulated, p < 0.01.

**Figure 3:**
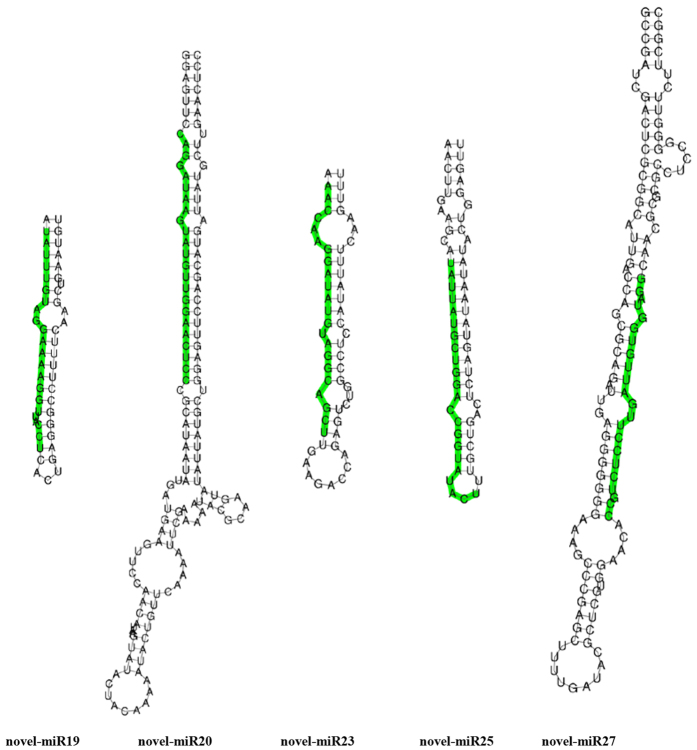
Precursor secondary structure prediction of five novel miRNAs differently expressed in Guiyan 1 (G) and Yunyan 2 (Y) in response to Cd stress. Green bars indicate the mature miRNAs, black letters show complementary base pairing (including mismatches).

Out of 164 miRNAs found in both genotypes, 72 miRNAs (27 families) were differentially expressed in response to Cd stress. Overall, 38, 116 and 10 miRNAs were up-regulated, unaltered and down-regulated, respectively, in response to Cd stress in Guiyan 1 with the corresponding figure for Yunyan 2 being 43, 106 and 15 (Fig. 2). Among these, 25 miRNAs (7 families: miR169, miR171, miR394, miR398, miR399, miR408 and miR482) were up-regulated while 8 miRNAs from a single family (miR166) and nta-miRNA6149a were down-regulated in both genotypes under Cd stress (Supplemental Table S4 and Table 2). In addition, 6 miRNA (nta-miR159, nta-miR319a, nta-miR319b, nta-miR396a, nta-miR6145e and nta-miR6149b) were unchanged in Yunyan 2 but up-regulated in Guiyan 1 under Cd stress (Table 3). In addition, 13 miRNAs (7 families such as miR156, miR169 and miR482) were down-regulated in Yunyan 2 but unchanged in Guiyan 1 under Cd stress (Table 3). Furthermore, 16 out of 30 novel miRNAs were equally expressed in all four libraries while the remaining 14 miRNAs were differentially expressed among the 4 miRNA libraries (Table 4). Also, the putative secondary structures of 5 novel miRNAs (i.e. novel-miR19, down-regulated in both genotypes under Cd stress; novel-miR20 and novel-miR23, only expressed in Guiyan 1; novel-miR25 and novel-miR27, unaltered in Guiyan 1 but down-regulated in Yunyan 2 under Cd stress) were predicted (Fig. 3).

### qRT-PCR validation

**Figure 4:**
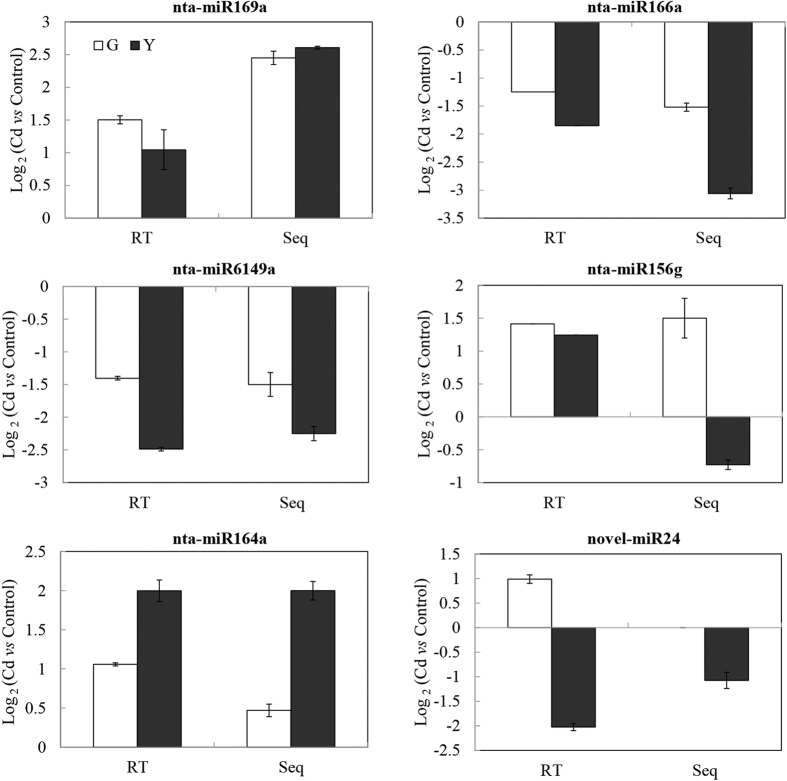
Validation expression patterns of six miRNAs identified in Guiyan 1 (G) and Yunyan 2 (Y) in response to Cd stress by qRT-PCR (RT). Seq represents high-throughput sequencing. The actin gene was used as a constitutive internal control for qRT-PCR.

To validate the high-throughput sequencing results, 6 miRNAs were randomly selected including 5 known and 1 novel miRNAs for qRT-PCR. As anticipated, the qRT-PCR results were consistent with the sequencing data (Fig. 4). For example, both the sequencing data and the qRT-PCR results showed that the expression levels of nta-miR166a and nta-miR6149a were down-regulated in Yunyan 2 but not changed in Guiyan 1 in response to Cd stress, and the expression level of nta-miR164a was prominently enriched in the Yunyan 2 under Cd stress. Although, the specific fold changes of nta-miR169a, nta-miR156g and novel-miR24 were not completely identical between the qRT-PCR and sequencing data, the miRNAs expression trends were similar between qRT-PCR and sequencing data in response to Cd stress.

### Target prediction and function analysis of miRNAs

**Figure 5:**
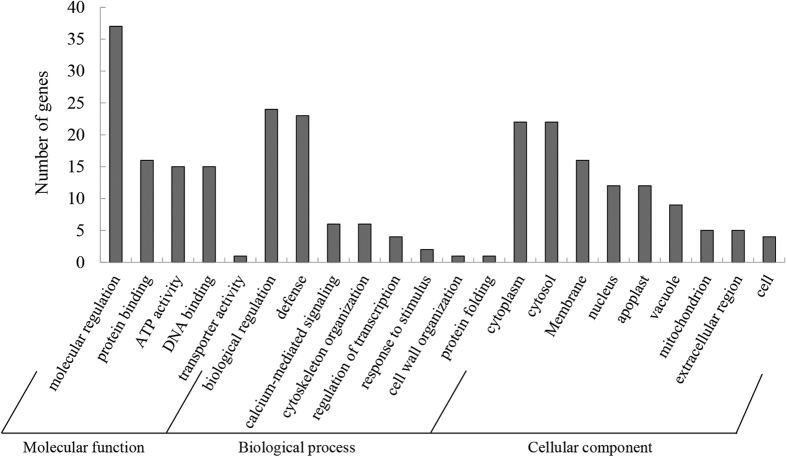
Gene ontology (GO) analysis of target genes of known and novel miRNAs identified in Guiyan 1 (G) and Yunyan 2 (Y). The y-axis indicates number of targeted genes in each GO category, the x-axis provides a definition of the GO terms.

Putative targets were predicted for the 164 known and 30 novel miRNAs by the web tool, psRNA Target (http://plantgrn.noble.org/psRNATarget/), using the tobacco DFCI gene index with 3 setting as the maximum expectation (Supplemental Table S5 and Supplemental Table S6). A total of 225 putative targets were predicted for 194 miRNAs (143 for known miRNAs and 87 for novel miRNAs). Five genes were targeted by both known and novel miRNAs. Of all the 194 miRNAs, miRNA nta-miR408 had the most potential targets (9) in the known miRNAs, and novel-miR30 had the most potential targets (9) in the novel miRNAs. Of all the 225 targets, the gene TC123361 had the highest number of potential miRNA regulators (6) in tobacco. To understand the biological function of miRNAs in tobacco, all putative target genes were subjected to GO functional classification by the GO slim tool of Blast2GO software (Fig. 5). Of all the putative target genes, 71 were assigned to the first-level of GO annotations of molecular function, biological process, and cellular location. The GO terms of ‘molecular regulation’ and ‘protein binding’ in molecular function, ‘biological regulation’ and ‘defense’ in biological process, ‘cytoplasm’ and ‘cytosol’ in cellular location accounted for more than 80% of all targeted genes analyzed (Fig. 5).

## Discussion

Tobacco is one of the most economically important crops worldwide. However, it is one of the susceptible plants to Cd stress, strategies for improving Cd tolerance in tobacco is urgently desired. In this study, we attempted to determine root microRNA profiles of two tobacco genotypes differing in Cd tolerance. Firstly, phenotypic responses of these two genotypes were compared under Cd stress. Although Yunyan 2 accumulated more Cd in shoots and roots than Guiyan 1, it showed better Cd-tolerance with a lower integrated score than Guiyan 1 under Cd stress, proving that it is more tolerant to Cd stress than Guiyan 1.

**Figure 6:**
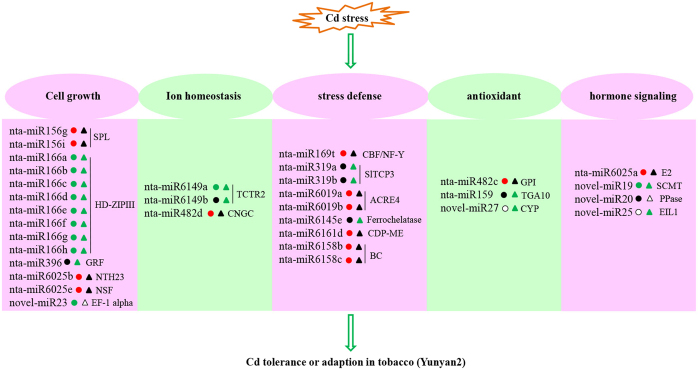
A hypothetically integrated schematic diagram of the mechanism involved in Cd tolerance in tobacco Yunyan 2. miRNAs labelled with red, black, green and white circles (Guiyan 1) and triangles (Yunyan 2) are up-regulated, unaltered, down-regulated and undetected in response to Cd stress, respectively. SPL, squamosa promoter binding like protein; GRF, growth-regulating factor; NSF, N-ethylmaleimide sensitive fusion protein; NTH23, *N. tabacum* homeobox 23; EF-1 alpha, elongation factor 1-alpha; CNGC, Cyclic nucleotide-gated calmodulin-binding ion channel; CBF/NF-Y, CCAAT-binding transcription factor; ACRE4, Avr9/Cf-9 rapidly elicited protein 4; CDP-ME, 4-diphosphocytidyl-2-C-methyl-D-erythritol kinase; BC, biotin carboxylase; GPI, glucose-6-phosphate isomerase; TGA10, bZIP factor; CYP, cytochromes P450; E2, ubiquitin carrier protein; PPase, serine/threonine protein phosphatase; SCMT, S-adenosyl-methionine-sterol-C-methyltransferase; EIL1, ethylene -stabilized transcription factor.

To explore the molecular mechanism of Cd tolerance in tobacco, miRNA expression profiles were compared between the two tobacco genotypes exposed to control and Cd stress conditions. From the length distribution of reads, we found that the length of miRNAs significantly altered by Cd stress, suggesting that miRNAs could be involved in the extensive regulation of gene expression in response to Cd stress in tobacco roots. In total, 85 differentially expressed miRNAs were identified, and miRNAs that showed different expression patterns between Guiyan 1 and Yunyan 2 were the focus for analysis. Based on the Cd responsive miRNAs identified in the two tobacco genotypes, we identified five areas (Fig. 6) of cell physiology where miRNAs appear to contribute to Cd tolerance and these are discussed below.

### miRNAs involved in cell growth

miRNAs have emerged as important players in the regulation of plant growth and development. miR166 can post-transcriptionally regulate class-III homeodomain-leucine zipper (HD-Zip III) transcription factors, which were demonstrated to be important for lateral root development^13^. miR156, which targets squamosal promoter binding like (SPL) transcription factors, is one of the most conserved and highly expressed miRNAs in plants, having an essential regulatory module in trichome development in *Arabidopsis*^14^. Huang *et al*.^15^ demonstrated that expression suppression of *NTH20* (a putative target of miR6025b) in tobacco leaves induced severe downward curling and abnormal growth of blades along the main veins^15^, and it has been reported that *N*-ethylmaleimide-sensitive fusion protein (a putative target of miR6025e) mediated membrane fusion and root hair tip growth in *Arabidopsis*^16^. Also, plant-specific growth-regulating factors (GRFs) are controlled by miR396, which have functions in leaf development^17^. In this study, 13 known miRNAs, i.e. nta-miR166a to nta-miR166h, nta-miR156g/i, nta-miR6025b/e and nta-miR396a, were detected as Cd-responsive miRNAs in tobacco. In addition, a novel miR23, that putatively targets elongation factor 1-alpha which is essential for regulation of the actin cytoskeleton and cell morphology^18^, was identified in the present study. All of the results imply that these miRNAs may be important post-transcriptional regulators involved in the regulation of tobacco root architecture, contributing to Cd tolerance in tobacco.

### miRNAs involved in ion homeostasis

Competition of Cd with essential metals for cellular uptake sites as well as binding sites in metalloenzymes disrupts homeostasis of essential elements. Cyclic nucleotide-gated channels (CNGCs) are Ca^2+^-permeable cation transport channels that are present in both animal and plant systems. They have been implicated in the uptake of both essential and toxic cations. Studies have revealed that CNGCs may function as a pathway for Ca^2+^ conduction into the cytosol as an early event during abiotic and biotic stresses^19^. In this study, nta-miR482d (targets CNGCs) was up-regulated in Guiyan 1 but unchanged in Yunyan 2 in response to Cd stress. And this might be a possible reason for the Cd-tolerant gene expression depends on Ca^2+^ signals in Yunyan 2 under Cd stress. Recent work showed that Cd stimulates Cu accumulation in roots of *Arabidopsis* and increases expression of genes encoding Cu transporters such as CTR1, CTR2 and CTRT6. Further analysis of Cd sensitivity of single and triple *ctr1ctr2ctr6* mutants and transgenic plants ectopically expressing CTR6 suggested that Cu uptake is an essential component of Cd resistance in *Arabidopsis*^20^. In this study, 2 miRNAs, nta-miRNA6149a (down-regulated in both genotypes) and nta-miRNA6149b (unchanged in Guiyan 1 but down-regulated in Yunyan 2) target CTR2 may contribute to ion balance for improving Cd tolerance in tobacco.

### miRNAs involved in stress defense

miRNA expression is differently regulated by several kinds of stresses. In response to drought stress, miR169 is down-regulated, whereas its target *NFYA5* (nuclear transcription factor Y subunit alpha, also known as the CCAAT-box binding factor) is induced in *Arabidopsis.* In addition, enhanced drought stress tolerance in transgenic creeping bentgrass is related to significant down-regulation of miR319 which targets *TCPs* (*TEOSINTE BRANCHED1/CYCLOIDEA/PROLIFERATING CELL FACTOR1*) encoding plant-specific transcription factors^21^,^22^. Silencing of an Avr9/Cf-9 rapidly elicited gene (an F-box gene with a Leu-rich-repeat domain) suppressed the hypersensitive response in tomato^23^ and might be regulated by miR6019. In this study, the endurance or resistance to Cd stress in Yunyan 2 might be enhanced by miR169 and miR6019 (up-regulated in Guiyan 1 but unchanged in Yunyan 2) and miR319 (unchanged in Guiyan 1 but down-regulated in Yunyan 2). Terpenoids, which constitute the most abundant and structurally diverse group of plant secondary metabolites, play an important role in plant-insect, plant-pathogen, and plant-plant interactions^24^. Silencing of two *Arabidopsis* ferrochelatase genes triggered different modes of plastid signaling in roots and leaves, enhancing salt stress responses^25^. Two miRNAs, nta-miR6161d (putatively targeting the 4-diphosphocytidyl-2-C-methyl-D-erythritol kinase gene for terpenoids synthesis) and nta-miR6145e (putatively targeting ferrochelatase), were detected in the current study. Plant responses to heavy metal stress are regulated by complex networks of miRNAs, indicating that the above differently expressed miRNAs identified in this study might response to Cd stress through negatively regulated their target genes in tobacco.

### miRNAs involved in redox maintenance

One of the inevitable consequences of Cd stress is enhanced reactive oxygen species (ROS) production in the different cellular compartments, disrupting the normal functions of plant cells^26^. It has been reported that the best characterized case study for redox-regulated signaling in plants is the TGA transcription factor (subfamily of basic leucine zipper transcription factors) pathway^27^. Seong *et al*.^28^ demonstrated that transient overexpression of the *Miscanthus sinensis* glucose-6-phosphate isomerase gene (*MsGPI*) in tobacco enhances expression of genes related to antioxidant metabolism^28^. The cytochromes P450 (*CYP*), a superfamily of heme-dependent enzymes are involved in the biosynthesis and detoxification of a wide variety of molecules^29^. In this study, three miRNAs, nta-miR159 (putatively targeting TGA10 transcription factor), nta-miR482 (putatively targeting glucose-6-phosphate isomerase) and novel-miR27 (putatively targeting cytochromes P450), were up-regulated in Guiyan 1 but unchanged in Yunyan 2 or unaltered in Guiyan 1 but down-regulated in Yunyan 2 under Cd stress. These results suggest that the Cd-tolerant, Yunyan 2, is more capable of scavenging Cd-induced ROS thereby reducing Cd toxicity in tobacco as a result of suppressing specific miRNAs.

### miRNAs involved in hormone signaling

Plant hormones can control plant development and stress responses through regulating different biological processes. In this study, three novel miRNAs, novel-miR19 (putatively targeting S-adenosyl-methionine-sterol-C-methyltransferase), novel-miR20 (putatively targeting serine/threonine protein phosphatases) and novel-miR25 (putatively targeting EIL1) were differently regulated by Cd stress. It has been reported that S-adenosyl-methionine-sterol-C-methyltransferase is an ethylene biosynthesis-related protein^30^. Serine/threonine protein phosphatase has multifaceted roles in regulating cellular signaling pathways, such as auxin and brassinosteroid signaling^31^, and Zhu *et al*.^32^ showed that jasmonate and ethylene signaling synergy can be mediated by derepressing the ethylene-stabilized transcription factor (EIN3/EIL1) in *Arabidopsis*^32^.

Based on the microRNA profile analysis of two tobacco genotypes of different Cd tolerance, 28 known miRNAs from 14 families and 5 novel miRNAs were identified as being linked with Cd tolerance in Yunyan 2. These miRNAs were mainly involved in processes associated with cell growth, ion homeostasis, stress defense, redox maintenance and hormone signaling. Regulation of these areas contributed to the better growth performance of Yunyan 2 than Guiyan 1 under Cd stress. In addition, the current results also provide some candidate miRNAs for further studies for the improvement of Cd tolerance in tobacco.

## Methods

### Plant materials and sample preparation

The greenhouse hydroponic experiment was carried out on Zijingang Campus, Zhejiang University, Hangzhou, China. Healthy tobacco (*Nicotiana tabacum* L.) seeds of Guiyan 1 (Cd-sensitive) and Yunyan 2 (Cd-tolerant) were germinated in sterilized, moist vermiculite in a growth chamber at 25 °C/20 °C (day/night). Uniform, healthy 4-leaf stage (50 d old) seedlings were transplanted to 5 L containers filled with 4.5 L basal nutrient solution (BNS), and the containers were placed in greenhouse. The composition of BNS was as follows: KNO_3_, 0.375 mM; CaNO_3_.4Η_2_Ο, 0.75 mM; MgSO_4_.7Η_2_Ο, 0.312 mM; KH_2_PO_4_, 0.25 mM; CuSO_4_.5Η_2_Ο, 0.025 μΜ; H_3_BO_3_, 6.25 μΜ; MnCl_2_.4Η_2_Ο, 0.5 μΜ; ZnSO_4_.7Η_2_Ο,0.5 μΜ; H_2_MoO_4_, 0.125 μΜ; ferric citrate, 10 μΜ^33^. The solution pH was adjusted to 5.8 ± 0.1 with HCl or NaOH as required. On the 7th day after transplanting, four treatments, G − Cd, G + Cd, Y − Cd and Y + Cd correspond to hydroponic tobacco Guiyan 1 grown in basic nutrition solution (BNS), Guiyan 1 in BNS + 50 μM Cd, Yunyan 2 in BNS and Yunyan 2 in BNS + 50 μM Cd, respectively, were commenced. The experiment was laid out in a split-plot design with treatment as the main plot and genotype as the sub-plot with five replicates for each treatment. The nutrient solution was continuously aerated with pumps. After 5 d of Cd exposure, fresh plant samples were directly used for the determination of growth parameters or dried for the measurement of Cd concentration. In addition, root samples were immediately frozen in liquid nitrogen and stored frozen at −80 °C for further analyses with 3 replicates.

### Chlorophyll content and growth measurement and Cd concentration analysis

After 5 d treatment, the upper second fully opened leaves of five plants from each treatment were selected to measure chlorophyll content as SPAD value using a chlorophyll meter (Minolta SPAD-502)^34^. After measuring plant heights and root lengths, roots were soaked in 20 mM Na_2_-EDTA for 3 h then rinsed thoroughly with deionized water. The plants were then separated into roots and shoots, and fresh weights measured. The roots and shoots were then dried at 80 °C and weighed. Dried roots and shoots were ground and ashed at 550 °C for 8 h, then digested with 30% HNO_3_. Cd concentrations were determined using flame atomic absorption spectrometry (Shimadzu AA-6300, Japan).

### RNA isolation, library preparation and sequencing of small RNAs

Total RNA was extracted using TRIzol reagent (Invitrogen, Carlsbad, CA, USA). Four small RNA libraries from the roots of plants from the G − Cd, G + Cd, Y − Cd and Y + Cd treatments were prepared based on Xu *et al*.^35^. Total RNA was separated using a 15% TBE-urea denaturing PAGE gel and small RNAs (10–35 nucleotides (nt)) were recovered. Then they were ligated to 5′-RNA adaptor (5′-GUUCAGAGUUCUACAGUCCGACGAUC-3′) and 3′-RNA adaptor (5′-pUCGUAUGCCGUCUUCUGCUUGUidT-3′) using T4 RNA ligase and, at each step length, were validated and purified by urea PAGE gel electrophoretic separation. The adapter-ligated small RNAs were subsequently transcribed into cDNA by Super-Script II Reverse Transcriptase (Invitrogen) and amplified by PCR. Then the amplified cDNA constructs were purified and recovered. After the amplification of cDNA, the sequencing libraries were sequenced using the Solexa’s proprietary sequencing-by-synthesis method^36^. The 1G sequencer, during automated extension cycles, recorded fluorophore excitation and determined the base sequence for each cluster.

### Bioinformatics analysis of small RNA and novel miRNA prediction

After trimming the adaptor sequences and removing low quality reads, small RNA sequences, fulfilling the Illumina pipeline quality control criteria, were compared with non-coding RNA sequences (tRNA, rRNA, snRNA and snoRNA) using Rfam (http://www.sanger.ac.uk/software/Rfam)^37^ and the GenBank database (http://www.ncbi.nlm.nih.gov)^38^. Sequences matching rRNA, tRNA, snRNA and snoRNA were removed. The remaining sequences were compared against 335,221 tobacco expressed sequence tags (ESTs) (http://www.ncbi.nlm.nih.gov/nucest/) and 1,420,595 genome survey sequences (GSSs) (http://www.ncbi.nlm.nih.gov/nucgss/) in the NCBI GenBank database using the CLC Genomics Workbench program 4.9. Finally, the clean, small RNA sequences were subjected to a BLASTn search with up to two mismatches against miRBase release 20.0 (http://www.mirbase.org/index.shtml). The leftover unannotated that perfectly matched with tobacco EST or GSS sequences were used to identify novel miRNAs by predicting precursors and their secondary structures using RNAfold (http://www.tbi.univie.ac.at/RNA/RNAfold.1.html)^39^ and analyzed by Mireap (http://sourceforge.net/projects/mireap/). All filtered small RNAs that could fold into a stem-loop structure were considered to be miRNAs.

### Differential expression of Cd-responsive miRNA

The frequency of miRNAs was normalized as transcripts per million (TPM), where TPM value = counts of this miRNA/total counts of this sample × 1000000. The fold change between the Cd-treated and control library from each of the two genotypes was calculated as: fold change = log_2_N, N = Cd(TPM)/control(TPM). The miRNAs with log_2_N ≥1.5 were up-regulated, between 0 < |log_2_N| < 1.5 were unchanged and log_2_N ≤ −1.5 were down-regulated, p < 0.01, respectively.

### Target genes prediction and function categories

The program, psRNATarget (http://plantgrn.noble.org/psRNATarget/), using default parameters, was used for the prediction of target mRNAs of tobacco known and novel miRNAs according to the established criteria^40^. In addition, function annotation of the predicted target genes by gene ontology (GO) terms was conducted using the GO slim tool in Blast2GO software (http://www.blast2go.org/).

### qRT-PCR validation

The hydroponic experiment was carried out again using Guiyan 1 and Yunyan 2 under control and 50 μM Cd treatments with 3 replicates. After 5 d Cd treatment, total RNAs were isolated from tobacco roots using TRIzol (Invitrogen, CA, USA) as described above. Small RNA was extracted using the mirVana miRNA Extraction Kit (Ambion, TX, USA). Quantitative real-time PCR (qRT-PCR) was performed using the CXF96 System (Bio-Rad, CA, USA) with a SYBR Green Supermix (Takara, Dalian, China) according to the manufacturer’s instructions. One of the uniformly expressed 5.8S rRNAs was used as the internal control for the stem-loop qRT-PCR. The PCR primer concentration was 10 μM. The reaction conditions were 95 °C for 30 s, followed by 40 cycles of 95 °C for 5 s and 60 °C for 10 s, and a melting curve analysis was generated to verify the specificity of the PCR amplification. Each experiment was replicated three times. The relative expression level was calculated using the 2^−ΔΔCT^ method^41^, and the fold change = log_2_ (2^−ΔΔCT^). The miRNAs with fold changes ≥1.5 or ≤−1.5 were considered to be up- or down-regulated in response to Cd stress, respectively. Stem-loop RT primers were designed according to previous work^42^,^43^, and were used for the reverse transcription of miRNA (Supplemental Table S7).

### Statistical analysis

Each result in this study was the mean of at least three replicates. Statistical analyses were performed with Data Processing System (DPS) statistical software package using ANOVA followed by Duncan’s multiple range tests (DMRT) to evaluate significant treatment effects at significance level of P ≤ 0.05.

## Additional Information

**How to cite this article**: He, X. *et al*. Identification and comparative analysis of the microRNA transcriptome in roots of two contrasting tobacco genotypes in response to cadmium stress. *Sci. Rep.* **6**, 32805; doi: 10.1038/srep32805 (2016).

## Supplementary Material

Supplementary Information

## Figures and Tables

**Table 1:** Summary of high-throughput sequencing of tobacco small RNAs from roots.

Library type	G − Cd	G + Cd	Y − Cd	Y + Cd
Total raw reads	6,931,266	7,453,307	6,219,546	6,553,483
Total clean reads	6,561,741(94.7)	6,945,431(93.2)	5,790,967(93.1)	5,631,677(85.9)
Unique clean reads	2,013,956(29.0)	1,394,044(18.7)	2,076,409(33.4)	1,061,349(16.2)
Total miRNA reads	358,704(5.2)	361,508(4.9)	367,226(6.0)	430,631(6.6)
Unique miRNA reads	24,295(0.4)	33,746(0.5)	22,921(0.4)	36,649(0.6)
Mapped to GSS				
Reads	2,178,186(31.4)	1,705,564(22.9)	2,604,528(41.9)	1,005,777(15.3)
Specific in each library	99,191(1.4)	40,451(0.5)	185,306(3.0)	255,39(0.4)
Conserved in all libraries	1,299,071(18.7)	1,392,321(18.7)	1,350,881(21.7)	838,234(12.8)
Unique reads	850,576(12.3)	386,030(5.2)	1,162,110(18.7)	221,124(3.4)
Specific in each library	96,742(1.4)	39,397(0.5)	179,792(3.0)	24,683(0.4)
Conserved in all libraries	290,456(4.2)	277,482(3.7)	325,681(5.2)	170,607(2.6)
Singleton	417,376(6.0)	311,391(4.2)	457,306(7.4)	216,938(3.3)
Mapped to EST				
Reads	1,602,311(23.1)	1,532,739(20.6)	1,765,463(28.4)	914,597(14.0)
Specific in each library	15,293(0.2)	13,575(0.2)	26,256(0. 4)	9,344(0.1)
Conserved in all libraries	1,450,188(20.9)	1,447,195(19.4)	1,542,202(24.8)	858,481(13.1)
Unique reads	423,852(6.1)	249,259(3.3)	561,780(9.0)	154,010(2.4)
Specific in each library	14,951(0.2)	13,187(0.2)	25,408(0.4)	9,059(0.1)
Conserved in all libraries	414,604(6.0)	315,876(4.2)	484,629(7.8)	185,894(2.8)
Singleton	843,150(12.2)	446,654(6.0)	1,056,345(17.0)	283,057(4.3)

Figure in parentheses represent the percentage of original reads. G − Cd, G + Cd, Y − Cd and Y + Cd correspond to hydroponically grown tobacco of the cultivars Guiyan 1 and Yunyan 2 grown in basic nutrition solution (BNS) or BNS + 50 μM Cd.

**Table 2:** Description, expression and putative target genes of known miRNAs whose expression were significantly down-regulated in roots of both Guiyan 1 (G) and Yunyan 2 (Y) treated with 50 μM Cd for 5 days.

Family name	miRNA name	Sequence	Length (nt)	TPM^a^				Fold change^b^		Target gene	Annotation
				G−Cd	G + Cd	Y−Cd	Y + Cd	G	Y		
miR166	nta-miR166a	UCGGACCAGGCUUCAUUCCCC	21	49481.3	17265.8	51937.0	6379.0	−1.5	−3.1	TC126889	PHAVOLUTA-like HD-ZIPIII protein (*N. sylvestris*)
	nta-miR166b	UCGGACCAGGCUUCAUUCCCC	21	49978.8	17303.7	51466.5	6467.4	−1.5	−3.0		
	nta-miR166c	UCGGACCAGGCUUCAUUCCCC	21	49723.1	17629.6	51621.0	6216.5	−1.5	−3.1		
	nta-miR166d	UCGGACCAGGCUUCAUUCCCC	21	49377.6	17569.0	51649.1	6725.6	−1.5	−3.0		
	nta-miR166e	UCGGACCAGGCUUCAUUCCCC	21	49636.7	17318.9	52232.0	6618.6	−1.5	−3.0		
	nta-miR166f	UCGGACCAGGCUUCAUUCCCC	21	50676.6	18054.1	52246.0	6390.7	−1.5	−3.1		
	nta-miR166g	UCGGACCAGGCUUCAUUCCCC	21	50369.1	17152.1	53095.9	6446.0	−1.6	−3.1		
	nta-miR166h	UCGGACCAGGCUUCAUUCCCC	21	49450.2	17091.5	53404.9	6560.9	−1.5	−3.1		
miR6149	nta-miR6149a	UUGAUACGCACCUGAAUCGGC	21	23011.6	8535.6	24061.7	5060.5	−1.5	−2.3	FS390832	TCTR2 protein (*S. lycopersicum*)

^a^TPM value indicates the expression level of miRNA; TPM value = counts of this miRNA/ total counts of this sample × 1000000.

^b^Fold change (Cd vs control) is log_2_N, log_2_N ≥ 1.5 are up-regulated, between 0 < |log_2_N| < 1.5 are unchanged and log_2_N ≤ −1.5 are down-regulated, p < 0.01. G–Cd, G + Cd, Y − Cd and Y + Cd correspond to hydroponically grown tobacco of the cultivars Guiyan 1 and Yunyan 2 grown in basic nutrition solution (BNS) or BNS + 50 μM Cd.

**Table 3:** Description, expression and putative target genes of known miRNAs whose expression were unchanged in Yunyan 2 (Y) but up-regulated in Guiyan 1 (G) roots, or down-regulated (−) in Yunyan 2 (Y) but unchanged in Guiyan 1 (G) roots treated with 50 μM Cd for 5 days.

Family name	miRNA name	Sequence	Length (nt)	TPM^a^				Fold change^b^		Target gene	Annotation
				G−Cd	G + Cd	Y−Cd	Y + Cd	G	Y		
miR156	nta-miR156g	UGACAGAAGAUAGAGAGCAC	20	317.8	897.3	955.2	575.1	1.5	−0.7	TC152836	Squamosa promoter binding like protein 12 (*Oryza sativa*)
	nta-miR156i	UGACAGAAGAUAGAGAGCAC	20	317.8	952.7	870.9	580.7	1.6	−0.6		
miR169	nta-miR169t	UAGCCAAGGAUGACUUGCCUU	21	925.9	2908.5	1826.0	4490.4	1.7	1.3	TC128216	CCAAT-binding transcription factor subunit B (*N. tabacum*)
										TC143413	
										TC167045	
										NP917237	
miR482	nta-miR482c	UUUCCAAUUCCACCCAUUCCUA	22	839.5	2476.3	962.2	2531.9	1.6	1.4	TC125874	Glucose-6-phosphate isomerase (*S. lycopersicum*)
										TC131822	
	nta-miR482d	UUCCCGACUCCCCCCAUACCAC	22	13818.7	53137.0	36640.4	95327.1	1.9	1.4	TC168260	Cyclic nucleotide-gated calmodulin-binding ion channel (*N. tabacum*)
miR6019	nta-miR6019a	UACAGGUGACUUGUAAAUGUUU	22	715.1	2459.1	1320.4	1770.4	1.8	0.4	FS424154	Avr9/Cf-9 rapidly elicited protein 4 (*N. tabacum*)
	nta-miR6019b	UACAGGUGACUUGUAAAUGUUU	22	677.1	2155.5	1116.7	1426.0	1.7	0.4		
miR6025	nta-miR6025a	UACCAACAAUUGAGAUAACAUC	22	894.8	3582.3	1524.0	2315.0	2.0	0.6	TC135300	Ubiquitin carrier protein (*Nicotiana tabacum*)
	nta-miR6025b	UGCCAACUAUUGAGAUGACAUC	22	666.8	2147.0	884.9	1614.2	1.7	0.9	TC124063	NTH23 protein (*N. tabacum*)
	nta-miR6025e	UGCCAAUUAUAGAGAUGACAUC	22	739.3	2139.2	1179.9	1384.9	1.5	0.2	TC123463	N-ethylmaleimide sensitive fusion protein (*N.tabacum*)
miR6158	nta-miR6158b	AAGUUCGAUUUGUACGAAGGGC	22	1402.6	3871.7	1474.9	3827.0	1.5	1.4	TC122996	Biotin carboxylase (*N. tabacum*)
	nta-miR6158c	AAGUUCGAUUUGUACGAAGGGC	22	1433.7	3949.0	1503.0	3964.5	1.5	1.4	FG150962	
miR6161	nta-miR6161d	UGAACUCCAGCAUAUUAUACU	21	863.7	2632.0	1812.0	2213.5	1.6	0.3	TC130660	4-diphosphocytidyl-2-C-methyl-D-erythritol kinase (*N. benthamiana*)
										TC142702	
										TC167586	
miR159	nta-miR159	UUUGGAUUGAAGGGAGCUCUA	21	49940.8	22774.7	41676.0	9735.2	−1.1	−2.1	TC148124	TGA10 transcription factor (*N. tabacum*)
										TC154362	
										EB446223	
										FG172155	
miR319	nta-miR319a	UUGGACUGAAGGGAGCUCCCU	21	7914.7	5074.6	17565.2	3011.8	−0.6	−2.5	TC150601	SlTCP3 (*S. lycopersicum*)
	nta-miR319b	UUGGACUGAAGGGAGCUCCCU	21	8246.3	4740.4	16919.0	2893.1	−0.8	−2.6		
miR396	nta-miR396a	UUCCACAGCUUUCUUGAACUG	21	6356.6	4040.3	11174.0	2444.9	−0.7	−2.2	TC131399	Growth-regulating factors (*N. tabacum*)
										AM814093	
										TC144468	
miR6145	nta-miR6145e	AUUGUUACAUGUAGCACUGGC	21	18468.7	11841.6	16954.2	5620.0	−0.6	−1.6	TC125028	Ferrochelatase (*N. tabacum*)
										TC140088	
										FG637935	
miR6149	nta-miR6149b	UUGAUACGCACCUGAAUCGGC	21	22994.3	8633.4	23563.0	5245.0	−1.4	−2.2	FS390832	TCTR2 protein (*S. lycopersicum*)

^a^TPM value = counts of this miRNA/ total counts of this sample × 1000000.

^b^Fold change (Cd vs control) is log_2_N, log_2_N ≥ 1.5 are up-regulated, between 0 < |log_2_N| < 1.5 are unchanged and log_2_N ≤ −1.5 are down-regulated, p < 0.01. G − Cd, G + Cd, Y − Cd and Y + Cd correspond to hydroponically grown tobacco of the cultivars Guiyan 1 and Yunyan 2 grown in basic nutrition solution (BNS) or BNS + 50 μM Cd.

**Table 4:** Description, expression and putative target genes of novel miRNAs from the roots of Guiyan 1 (G) and Yunyan 2 (Y) treated with 50 μM Cd for 5 days.

miRNA name	Sequence	Length (nt)	TPM^a^				Fold change^b^		Target gene	Annotation
			G − Cd	G + Cd	Y − Cd	Y + Cd	G	Y		
novel-miR1	ATCATGCTATCCCTTTGGACT	21	122.8	123.2	129.5	130.5	1.0	1.0	TC156152	CC-NBS-LRR protein (*S. tuberosum*)
									TC156159	
									AM844322	
									TC166323	
									FS375002	
novel-miR2	GGAATGTTGTCTGGCTCGAGG	21	37.5	37.5	41.4	39.6	1.0	0.0	TC141351	Guanine nucleotide-binding protein subunit beta-2 (*N. tabacum*)
									TC144685	
									TC147733	
novel-miR3	CTAGAACTCCAGCATAATATACT	23	9.8	10.1	10.4	9.8	1.0	−0.1	TC126710	EBP6 (*N. tabacum*)
novel-miR4	TCCACATCCTTGTTGATAACTG	22	9.1	8.8	9.7	9.1	0.0	−0.1	TC123427	EIL5 (*N. tabacum*)
									FG137480	
									TC126725	
									FG163120	
novel-miR5	TTGTGAGACAAAAAGAAGCCT	21	6.2	6.4	6.6	6.5	1.0	0.0	TC127378	G6PD (*N. tabacum*)
novel-miR6	TCCAAAGGGATCGCATTGATC	21	5.5	5.8	5.5	5.7	0.9	1.0	TC133182	Transport inhibitor response 1 (*G. hirsutum*)
									TC148720	
novel-miR7	ATTTGGTCTAGTGGTATGATTCT	23	4.3	4.4	4.7	4.6	1.0	0.0	TC122895	CYP (*N. tabacum*)
									TC125350	
									FG640560	
novel-miR8	TTGGTGATATTTCTTCGGATT	21	4.0	5.2	4.1	5.4	0.8	0.8	TC123057	Villin 2 (*N. tabacum*)
									TC131622	
novel-miR9	TTCTTTTGGACAAGTAGCACC	21	3.0	3.1	3.1	3.1	1.0	1.0	AM810590	DEAD-box ATP-dependent RNA helicase 37 (*O. sativa*)
novel-miR10	CATAGCCAATCTTTGGAGCCT	21	2.3	2.3	2.4	2.4	1.0	0.0	TC124312	ASR4 (*S. lycopersicum*)
									TC146505	
									EB428262	
									TC127090	DS2 protein (*S. tuberosum*)
									FS433531	
									TC125178	Abscisic stress ripening protein (*S. chacoense*)
novel-miR11	TATCGGTTTAGCTCTTATCGGGC	23	1.5	1.5	1.6	1.7	1.0	0.9	FG185054	Expansin-like protein precursor (*S. lycopersicum*)
novel-miR12	CTGGGTGGTGTAGTCGGTTATC	22	1.4	1.4	1.6	1.6	1.0	1.0	TC134073	Extensin (*N. tabacum*)
									TC145834	
									TC169113	
									DW002761	
novel-miR13	TTAACTTTTGAACTTGGAACTCA	23	1.2	1.3	1.4	1.2	0.9	−0.1	TC124856	SGT1 (*N. benthamiana*)
									TC161731	
									FG142982	
novel-miR14	GATACATGTGTCGCAGAAGACTT	23	1.2	1.3	1.4	1.3	0.9	−0.1	TC130797	CDC5-like protein (*S. lycopersicum*)
									TC130688	
novel-miR15	TGAAGAAGAATGAACTAGCACC	22	1.1	1.1	1.2	1.2	1.0	1.0	HS085450	F28C11.9 (*A. thaliana*)
novel-miR16	AATCCGAGCCCCACATTCATC	21	0.9	1.0	0.9	0.9	0.9	1.0	TC127696	Phi-1 protein (*N. tabacum*)
novel-miR17	CGTCTCCTTGATTGTGGTAGGC	22	3.2	3.4	0.0	3.3	—	↑	TC141992	Actin-related protein 3 (*N. tabacum*)
									FS430341	
novel-miR18	AAGGATTCAAGGTAGAGCTGCTT	23	1.8	1.9	0.0	1.1	—	↑	TC126568	IAA9 protein (*N. tabacum*)
									TC151478	
novel-miR19	TATTTGTAGGAAAAGGTTACCT	22	1.5	0.0	1.7	0.0	↓	↓	TC126513	S-adenosyl-methionine-sterol-C-methyltransferase homolog (*N. tabacum*)
									TC134252	
novel-miR20	CAGGATAAGTATGTTGGAACTCC	23	1.5	0.7	0.0	0.0	—	—	FG155555	Serine/threonine protein phosphatase (*S. lycopersicum*)
									FG195049	
novel-miR21	ACGAGGTTCGGACAAGTTGCA	21	0.8	0.8	0.0	0.4	—	↑	FG191056	PttA (*Petunia* x *hybrida*)
novel-miR22	CAACATGTGGAAGATCTTAGCA	22	0.8	0.8	0.0	0.9	—	↑	AM815929	WRKY transcription factor (*N. tabacum*)
									FG193928	
novel-miR23	AACCAAGGATATGTAGGCAGCT	22	0.8	0.0	0.0	0.0	↓	—	TC123450	Elongation factor 1-alpha (*N. tabacum*)
									TC123811	
									TC124472	
									FG141704	
novel-miR24	TCCAGCGGCTGGAAGAGCAC	20	0.0	0.0	18.3	8.7	—	—	TC128664	Ethylene receptor ERS homolog (*N. tabacum*)
									TC147271	
									TC148676	
									FS427700	
									FS406072	
novel-miR25	TATTATGCTGGACCGGTATACT	22	0.0	0.0	3.6	0.0	—	↓	FG162393	EIL1 (*Petunia* x *hybrida*)
									FG170485	
novel-miR26	TGAGTGTGAGGCGTTGGATTGA	22	0.0	2.8	3.1	3.0	↑	—	TC131218	Glucose-1-phosphate adenylyltransferase (*N. tabacum*)
									TC140583	
									TC123361	
									TC129146	
novel-miR27	CCGTCTCCTTGATTGTGGTAGG	22	0.0	0.0	3.1	0.0	—	↓	TC124278	Cytochromes P450 (*N. tabacum*)
									TC122897	
									FG165245	
									TC122880	
									TC130596	
									TC160946	
novel-miR28	ATAATATACTGGAGATTGGAGCC	23	0.0	0.0	0.9	0.4	—	—	TC147905	Extensin 1 (*N. tabacum*)
novel-miR29	AGAGAGACTGTTTCCGATAGACC	23	0.0	0.0	0.0	7.0	—	↑	TC123392	Eukaryotic initiation factor (*N. tabacum*)
									TC124140	
									EB440487	
									FG190354	
									TC122931	
novel-miR30	GTGAGCATACCTGTCGGGACCC	22	0.0	0.0	0.0	12.7	—	↑	TC159027	Vacuolar ATP synthase 16 kDa proteolipid subunit (*N. tabacum*)
									TC124894	
									TC127633	
									TC123167	
									TC127603	
									TC138401	
									TC167606	
									FG633653	
									FS406852	

^a^TPM value indicates the expression level of miRNA; TPM value = counts of this miRNA/ total counts of this sample × 1000000.

^b^Fold change (Cd vs control) is log_2_N, log_2_N ≥ 1.5 are up-regulated, between 0 < |log_2_N| < 1.5 are unchanged and log_2_N ≤ −1.5 are down-regulated, p < 0.01. “↑” indicates only expressed in Cd-treated, “↓” only expressed in control, “—” not expressed in both conditions or unchanged. G − Cd, G + Cd, Y − Cd and Y + Cd correspond to hydroponically grown tobacco of the cultivars Guiyan 1 and Yunyan 2 grown in basic nutrition solution (BNS) or BNS + 50 μM Cd.
