# Supplementary material for: Identification and comparative analysis of the microRNA transcriptome in roots of two contrasting tobacco genotypes in response to cadmium stress
Source: Sci Rep. 2016 Sep 26;6:32805. doi: 10.1038/srep32805 (PMC5036098; doi:10.1038/srep32805)
Supplement: Supplementary Information [file srep32805-s1.pdf]

Identification and comparative analysis of the microRNA transcriptome in roots of two contrasting tobacco genotypes in response to cadmium stress

Xiaoyan He, Weite Zheng, Fangbin Cao, Feibo Wu*

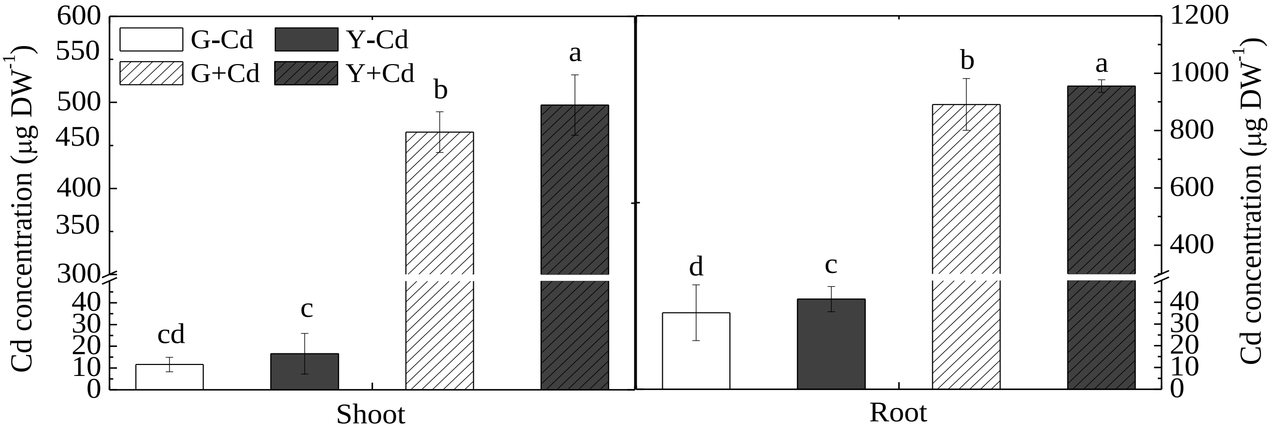

Figure S1. Cd concentrations in shoots and roots of Guiyan 1 (G) and Yunyan 2 (Y) under 50 μM Cd stress for 5 days. Error bars represent SD values (n=3). Different letters indicate significant differences ($P<0.05$) among each treatment of the two genotypes. G–Cd, G+Cd, Y-Cd and Y+Cd correspond to hydroponically grown tobacco of the cultivars Guiyan 1 and Yunyan 2 grown in basic nutrition solution (BNS) or BNS + 50 μM Cd.

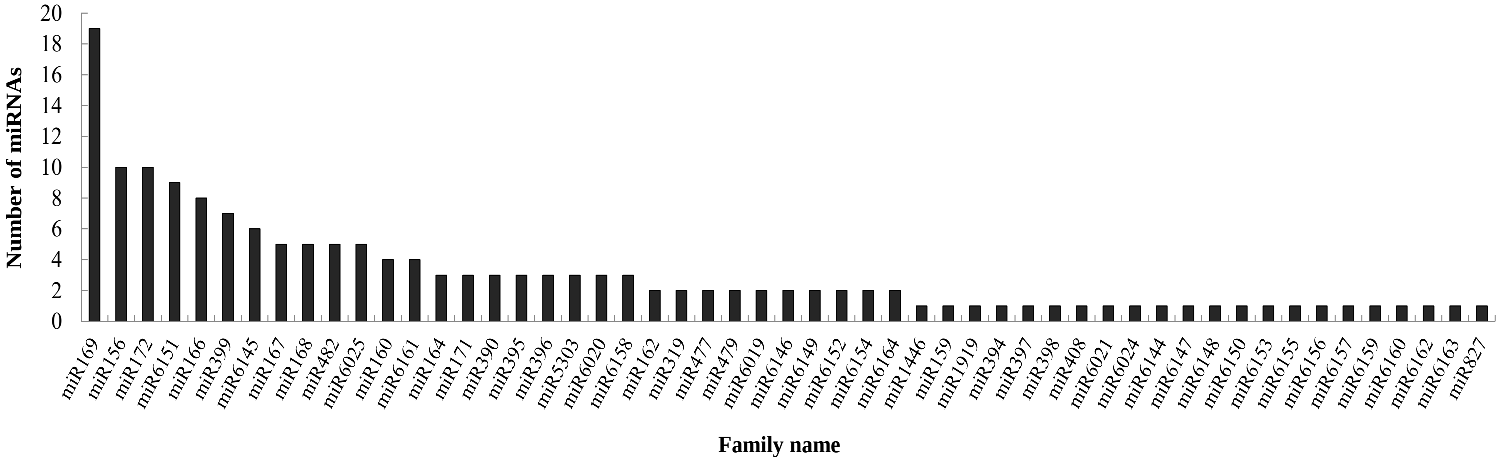

Figure S2. Family size (number of miRNAs in each family) of the known miRNAs in tobacco roots.

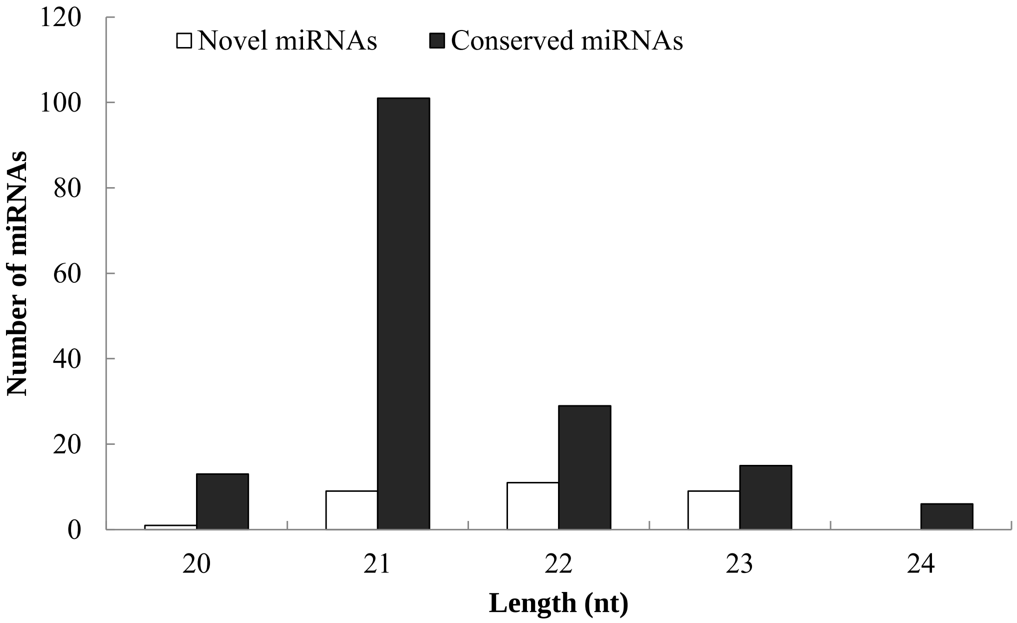

Figure S3. Length based distribution of known and novel miRNAs in tobacco roots.

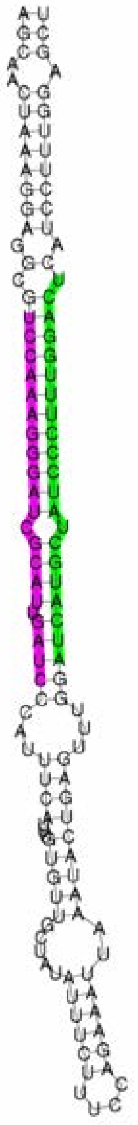

ATCATGCTATCCCTTTGGACT_Niben044Scf00002137_350188_32708-32728

novel-miR1

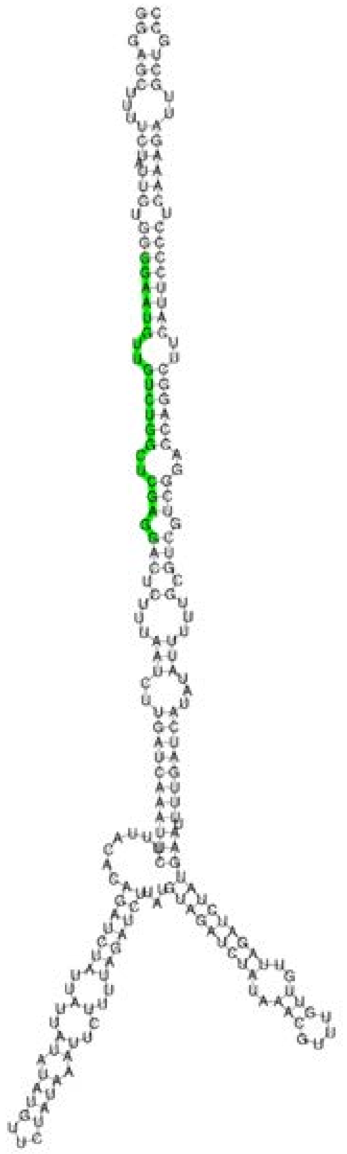

GGAATGTTGTCTGGCTCGAGG_Niben044Scf00014823_335993_245585-245605

novel-miR2

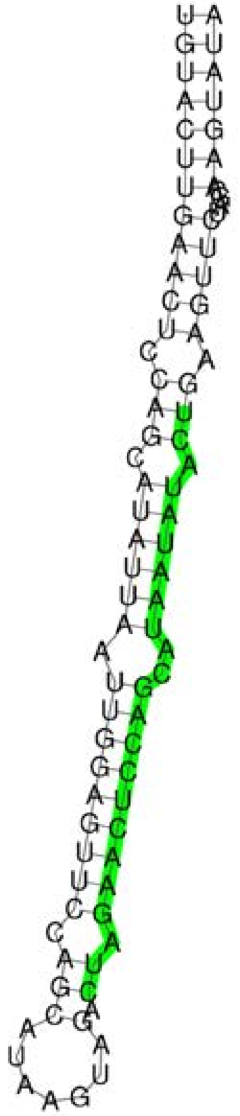

CTAGAACTCCAGCATAATATACT_Niben044Scf00004045_395210_262611-262633

novel-miR3

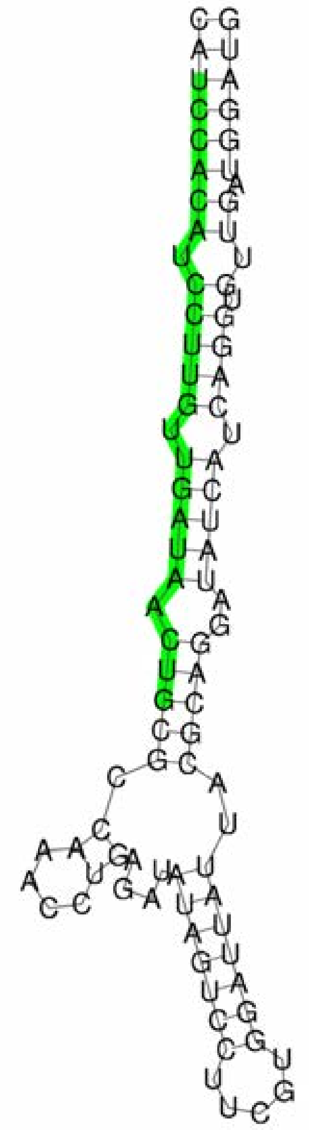

TCCACATCCTTGTTGATAACTG_Niben044Scf00004103_360652_102876-102897

novel-miR4

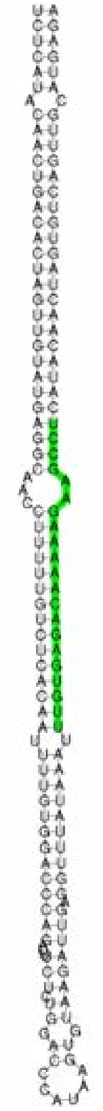

TTGTGAGACAAAAAGAAGCCT_Niben044Scf00023006_384368_116423-116443

novel-miR5

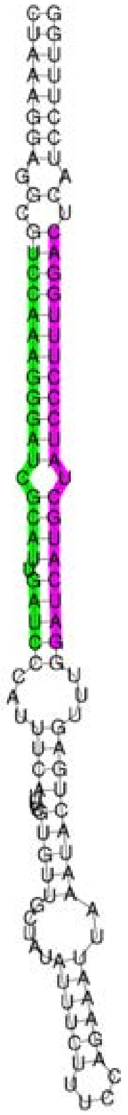

TCCAAAGGGATCGCATTGATC_Niben044Scf00002137_350188_32634-32654

novel-miR6

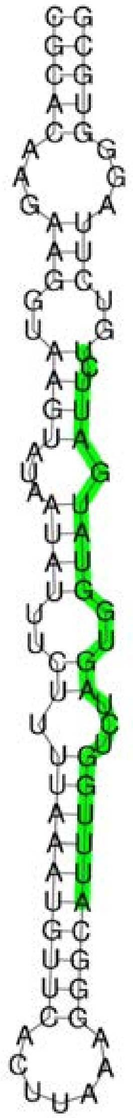

ATTTGGTCTAGTGGTATGATTCT_Niben044Scf00012994_341128_316112-316134

novel-miR7

novel-miR8

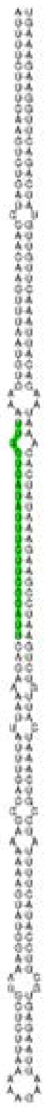

TTGGTGATATTTCTTCGGATT_Niben044Scf00001682_380483_208795-208815

novel-miR8

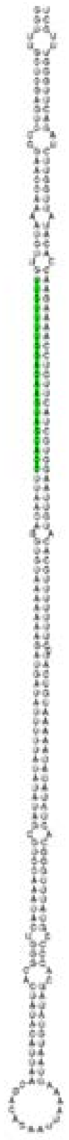

TTCTTTTGGACAAGTAGCACC_Niben044Scf00006151_355967_99846-99866

novel-miR9

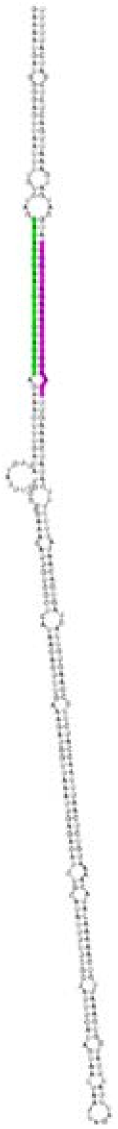

CATAGCCAATCTTTGGAGCCT_Niben044Scf00017997_399588_109107-109127

novel-miR10

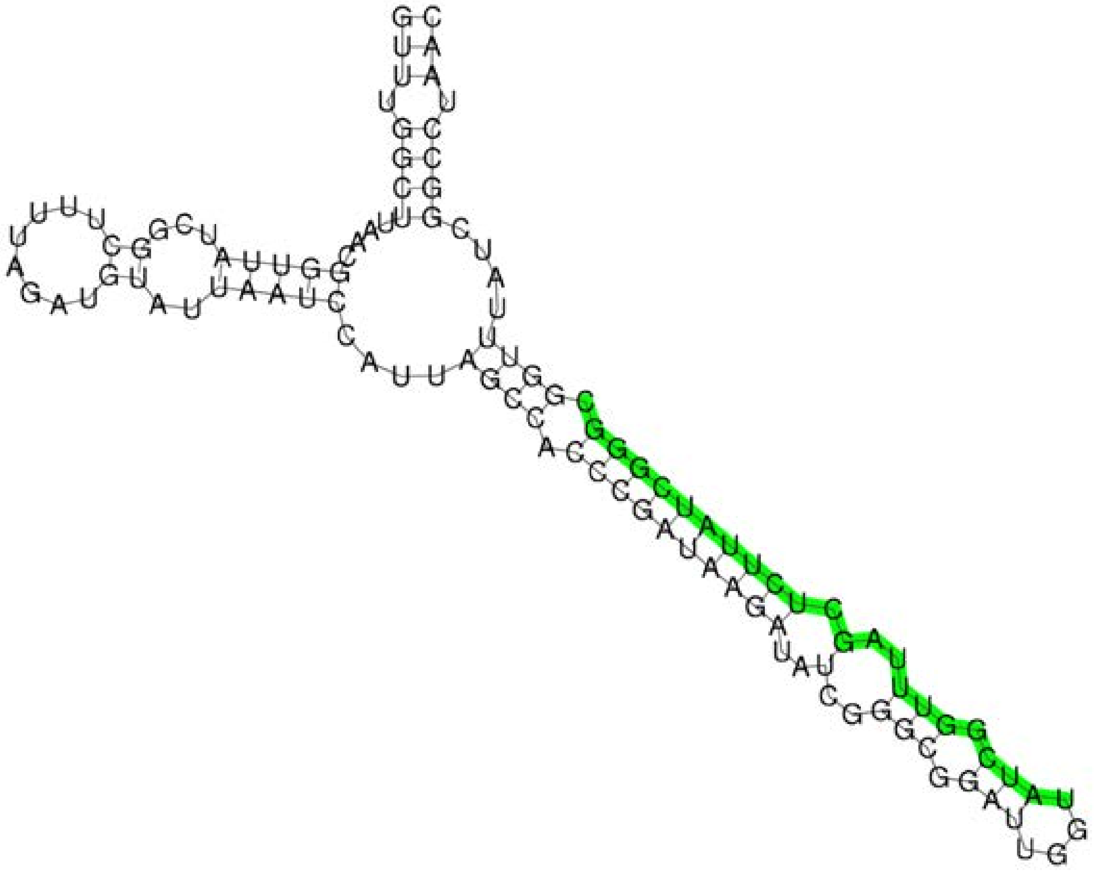

TATCGGTTTAGCTCTTATCGGGC_Niben044Scf00002443_315754_205534-205556

novel-miR11

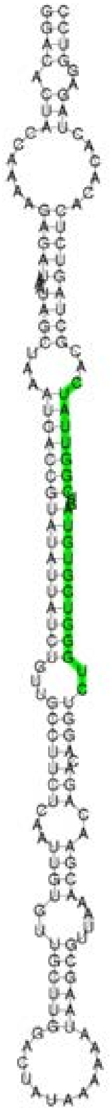

CTGGGTGGTGTAGTCGGTTATC_Niben044Scf00009074_604417_219730-219751

novel-miR12

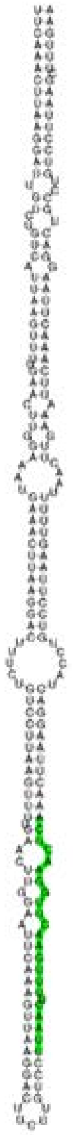

TTAACTTTTGAACTTGGAACTCA_Niben044Scf00006338_477699_443234-443256

novel-miR13

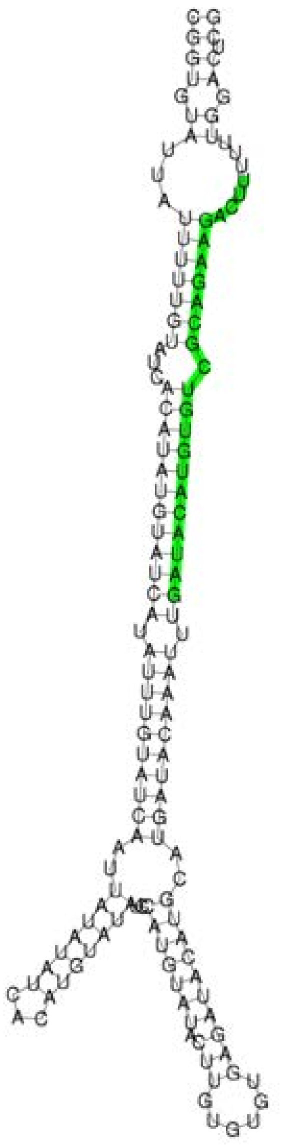

GATACATGTGTCGCAGAAGACTT_Niben044Scf00002388_322526_310689-310711

novel-miR14

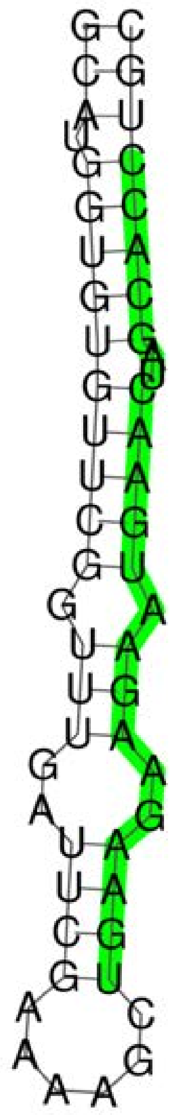

TGAAGAAGAATGAACTAGCACC_Niben044Scf00003359_572295_508425-508446

novel-miR15

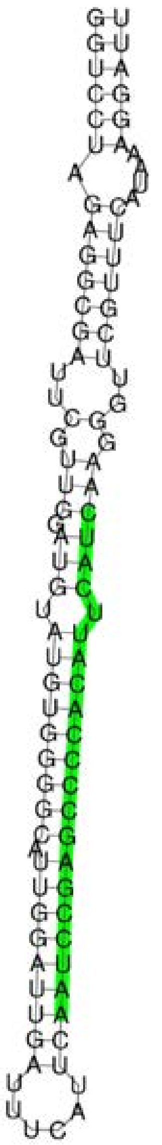

AATCCGAGCCCCACATTCATC_Niben044Scf00002583_361192_317931-317951

novel-miR16

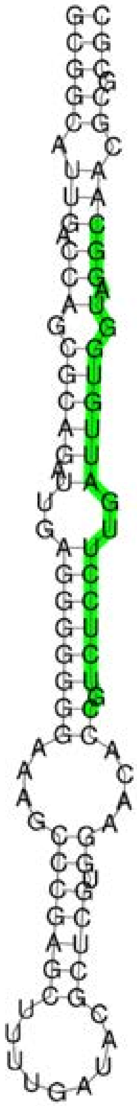

CGTCTCCTTGATTGTGGTAGGC_Niben044Scf00003611_445722_318844-318865

novel-miR17

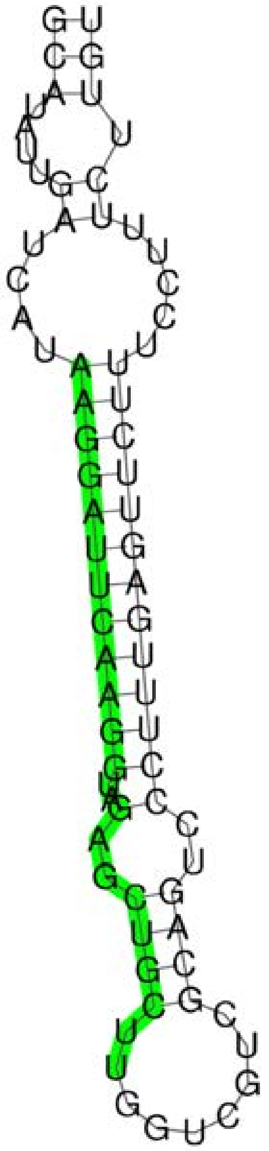

AAGGATTCAAGGTAGAGCTGCTT_Niben044Scf00004802_433360_238081-238103

novel-miR18

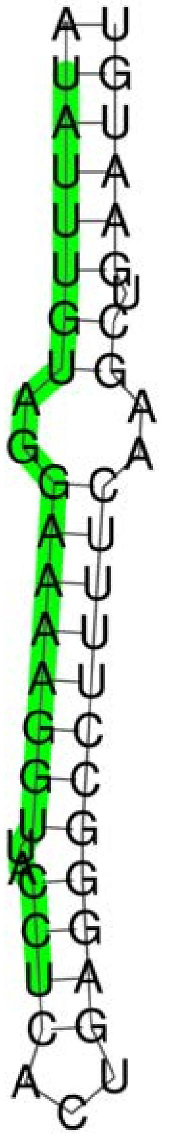

TATTTGTAGGAAAAGGTTACCT_Niben044Scf00000377_326229_90708-90729

novel-miR19

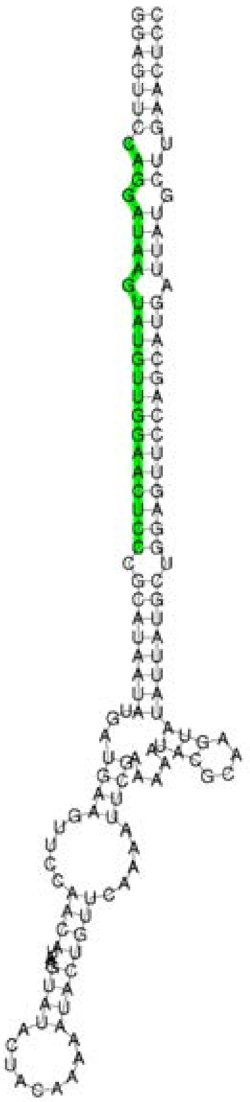

CAGGATAAGTATGTTGGAACTCC_Niben044Scf00002190_346493_112848-112870

novel-miR20

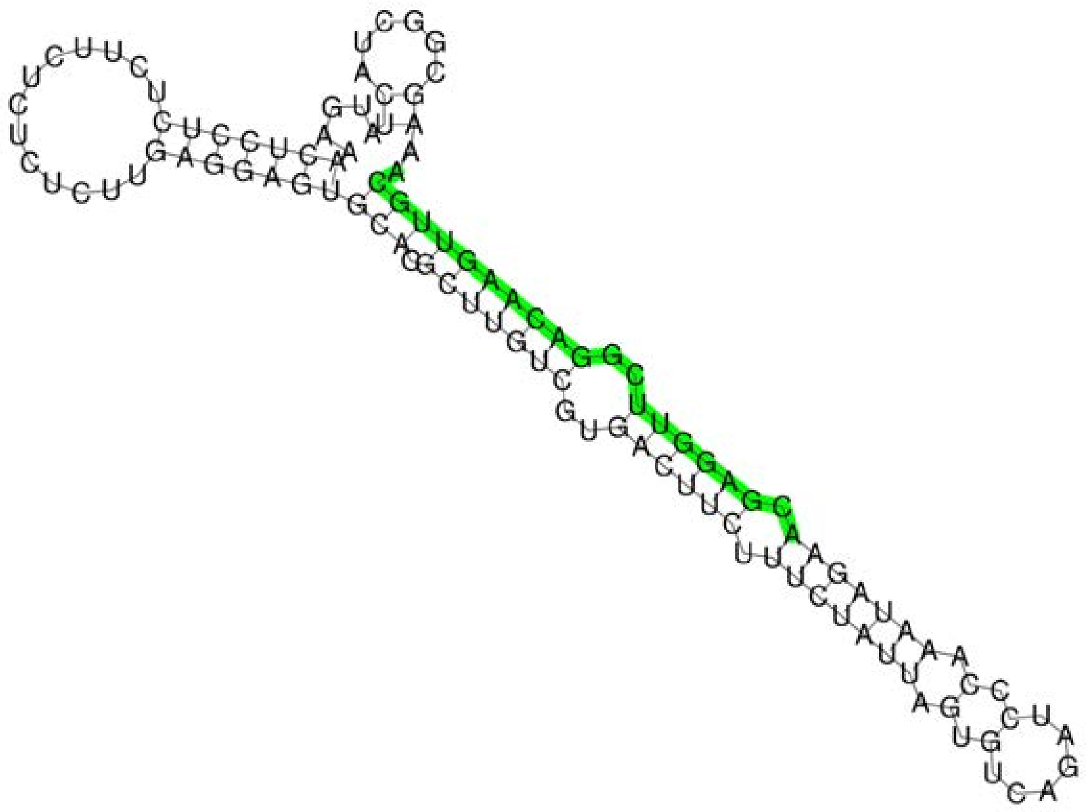

ACGAGGTTCGGACAAGTTGCA_Niben044Scf00019316_317706_167182-167202

novel-miR21

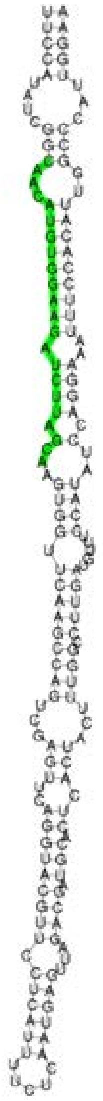

CAACATGTGGAAGATCTTAGCA_Niben044Scf00003134_352543_336479-336500

novel-miR22

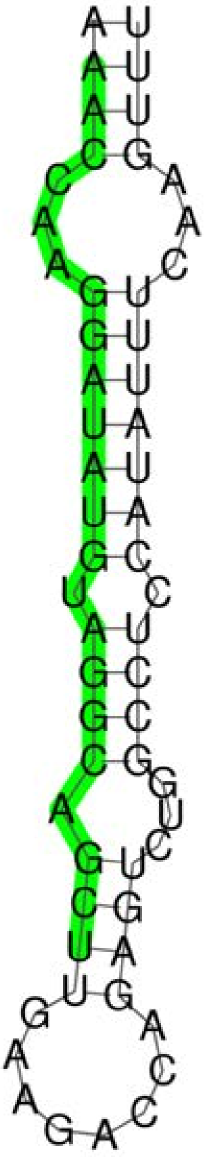

AACCAAGGATATGTAGGCAGCT_Niben044Scf00016243_362612_196130-196151

novel-miR23

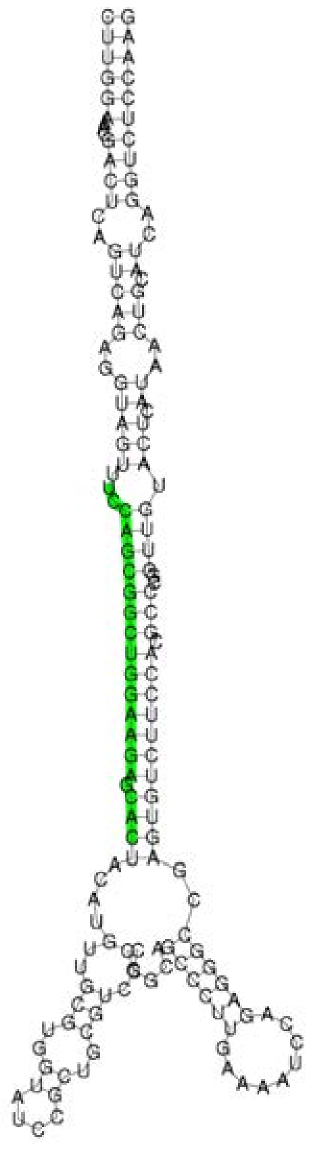

TCCAGCGGCTGGAAGAGCAC_Niben044Scf00022866_467875_216492-216511

novel-miR24

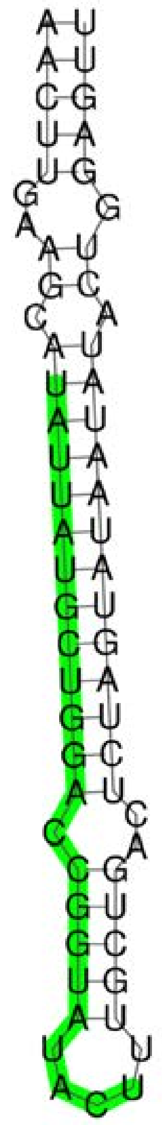

TATTATGCTGGACCGGTATACT_Niben044Scf00004550_314476_73035-73056

novel-miR25

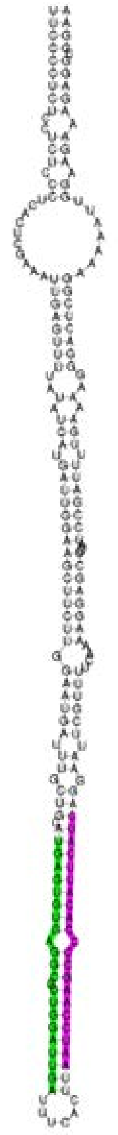

TGAGTGTGAGGCGTTGGATTGA_Niben044Scf00009193_366732_292363-292384

novel-miR26

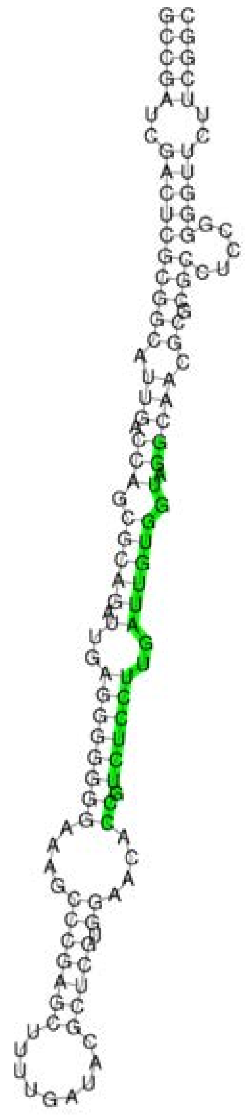

CCGTCTCCTTGATTGTGGTAGG_Niben044Scf00003611_445722_318843-318864

novel-miR27

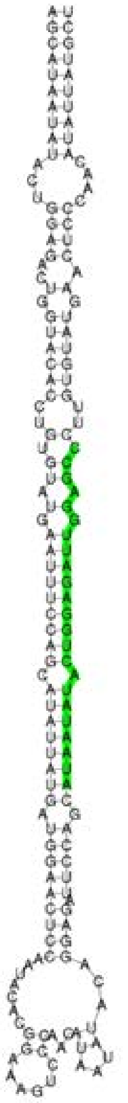

ATAATATACTGGAGATTGGAGCC_Niben044Scf00012719_341801_236835-236857

novel-miR28

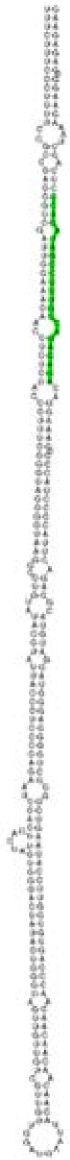

AGAGAGACTGTTTCCGATAGACC_Niben044Scf00013296_316665_20878-20900

novel-miR29

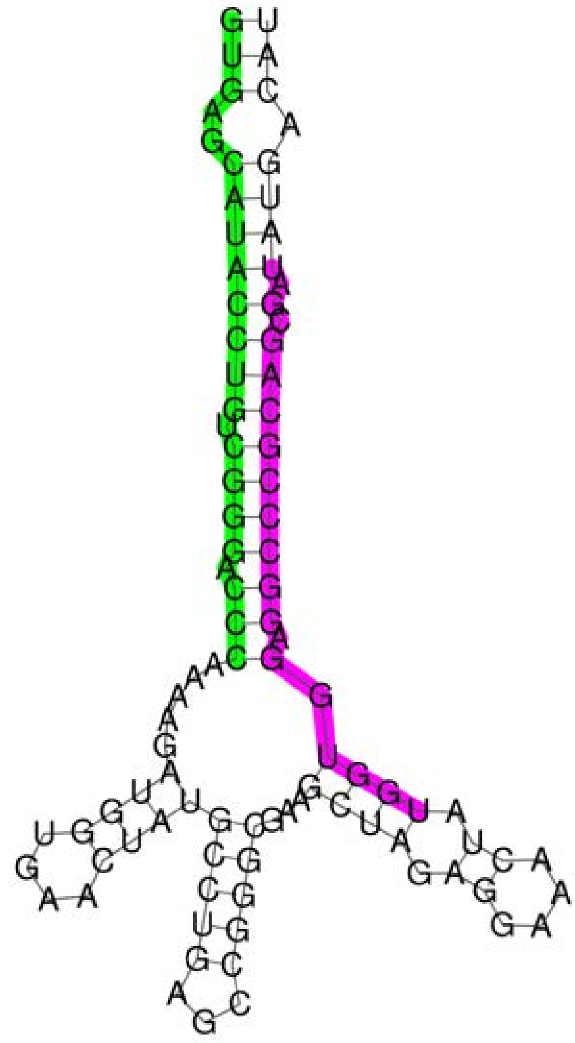

GTGAGCATACCTGTCGGGACCC_Niben044Scf00003611_445722_317845-317866

novel-miR30

Figure S4. Precursor secondary structure prediction of 30 tobacco novel miRNAs. Green bars indicate the mature miRNAs located in the 3' ends, pink bars indicate mature miRNAs located in the 5' ends, black letters show complementary base pairing (including mismatches).

Table S1 Effects of Cd on the growth and chlorophyll contents of Guiyan 1 (G) and Yunyan 2 (Y) under control and 50 μM Cd conditions at day 5.

Genotype	Plant height (cm)	Root length (cm)	Fresh weight (g)		Dry weight (g)		SPAD	Integrated score*
			Shoot	Root	Shoot	Root		
G-Cd	5.167 a	7.750 b	0.500ab	0.123 a	0.036 b	0.008 ab	20.733 a	G=43.173
G+Cd	3.733 c	5.500 c	0.285 d	0.065 c	0.021 d	0.004 c	8.264 c	
Y-Cd	5.250 a	8.100 a	0.525 a	0.120 a	0.044 a	0.009 a	20.443 a	Y=35.077
Y+Cd	4.400 b	5.500 c	0.345 c	0.100 b	0.027 c	0.004 c	9.313 b	

* Integrated score = absolute values of [SPAD value[a] × 0.1429 + plant height[a] × 0.1429 + root length[a] × 0.1429 + fresh weight[a] × 0.1429 + dry weight[a] × 0.1429]

([a]reduced (-)/increased (+) percentage in growth/physiological parameters relative to the controls)
Different letters indicate significant differences (P<0.05) among treatments within two genotypes.
G–Cd, G+Cd, Y-Cd and Y+Cd correspond to hydroponic tobacco Guiyan 1 grown in basic nutrition solution (BNS), Guiyan 1

Table S2 Known miRNAs expressed in both tobacco genotypes Guiyan 1 (G) and Yunyan 2 (Y) under control and 50 μM Cd conditions at day 5.

Family name	miRNA name	Sequence	Length (nt)	TPM[a]				Fold change[b]		P-value	
				G-Cd	G+Cd	Y-Cd	Y+Cd	G	Y	G	Y
miR1446	nta-miR1446	UGAACUCUCUCCCUCAAUGGCU	22	2566.8	5477.6	3020.0	3332.9	1.1	0.1	4.7E-232	0.005392
miR156	nta-miR156a	UGACAGAAGAGAGUGAGCAC	20	587.3	786.2	597.0	782.4	0.4	0.4	1.15E-07	1.84E-05
	nta-miR156b	UGACAGAAGAGAGUGAGCAC	20	518.2	912.8	449.5	856.4	0.8	0.9	5.45E-14	5.1E-24
	nta-miR156c	UGACAGAAGAGAGUGAGCAC	20	449.1	880.7	519.7	858.4	1.0	0.7	1.1E-20	2.26E-13
	nta-miR156d	UGACAGAAGAGAGUGAGCAC	20	573.5	878.6	477.6	815.9	0.6	0.8	1.39E-11	1.87E-16
	nta-miR156e	UGACAGAAGAGAGUGAGCAC	20	563.1	856.2	414.4	851.1	0.6	1.0	3.69E-13	1.79E-29
	nta-miR156f	UGACAGAAGAGAAUGAGCAC	20	639.1	1117.7	337.1	829.7	0.8	1.3	2.65E-23	2.5E-40
	nta-miR156g	UGACAGAAGAUAGAGAGCAC	20	317.8	897.3	955.2	575.1	1.5	-0.7	2.61E-43	3.46E-20
	nta-miR156h	UGACAGAAGAUAGAGAGCAC	20	342.0	889.7	863.9	530.8	1.4	-0.7	1.01E-35	9.25E-14
	nta-miR156i	UGACAGAAGAUAGAGAGCAC	20	317.8	952.7	870.9	580.7	1.6	-0.6	6.55E-68	4.27E-13
	nta-miR156j	UGACAGAAGAUAGAGAGCAC	20	369.7	923.8	948.1	573.1	1.3	-0.7	1.24E-39	9.53E-22
miR159	nta-miR159	UUUGGAUUGAAGGGAGCUCUA	21	49940.8	22774.7	41676.0	9735.2	-1.1	-2.1	0	0
miR160	nta-miR160a	UGCCUGGCUCCCUGUAUGCCA	21	3700.0	3697.0	1222.0	3261.2	0.0	1.4	0.056199	4.1E-190
	nta-miR160b	UGCCUGGCUCCCUGUAUGCCA	21	3731.0	3969.3	1467.9	3403.5	0.1	1.2	0.098122	3.7E-156
	nta-miR160c	UGCCUGGCUCCCUGUAUGCCA	21	3772.5	3848.6	1264.2	3306.3	0.0	1.4	0.385231	1.4E-180
	nta-miR160d	UGCCUGGCUCCCUGCAUGCCA	21	5019.6	5041.9	1938.4	4733.6	0.0	1.3	0.633527	1.6E-247
miR162	nta-miR162a	UCGAUAAACCUCUGCAUCCAG	21	1340.4	1747.3	1601.3	2606.5	0.4	0.7	4.29E-06	4.92E-45
	nta-miR162b	UCGAUAAACCUCUGCAUCCAG	21	1388.8	1970.1	1699.6	2571.6	0.5	0.6	7.1E-18	1.17E-22
miR164	nta-miR164a	UGGAGAAGCAGGGCACGUGCA	21	3869.2	5366.1	1460.8	5850.5	0.5	2.0	5.7E-42	0
	nta-miR164b	UGGAGAAGCAGGGCACGUGCA	21	3869.2	5525.0	1573.2	5738.8	0.5	1.9	7.75E-48	0
	nta-miR164c	UGGAGAAGCAGGGCACAUGCU	21	3530.7	4833.4	1720.7	4856.8	0.5	1.5	1.09E-36	0
miR166	nta-miR166a	UCGGACCAGGCUUCAUUCCCC	21	49481.3	17265.8	51937.0	6379.0	-1.5	-3.1	0	0
	nta-miR166b	UCGGACCAGGCUUCAUUCCCC	21	49978.8	17303.7	51466.5	6467.4	-1.5	-3.0	0	0
	nta-miR166c	UCGGACCAGGCUUCAUUCCCC	21	49723.1	17629.6	51621.0	6216.5	-1.5	-3.1	0	0
	nta-miR166d	UCGGACCAGGCUUCAUUCCCC	21	49377.6	17569.0	51649.1	6725.6	-1.5	-3.0	0	0
	nta-miR166e	UCGGACCAGGCUUCAUUCCCC	21	49636.7	17318.9	52232.0	6618.6	-1.5	-3.0	0	0
	nta-miR166f	UCGGACCAGGCUUCAUUCCCC	21	50676.6	18054.1	52246.0	6390.7	-1.5	-3.1	0	0
	nta-miR166g	UCGGACCAGGCUUCAUUCCCC	21	50369.1	17152.1	53095.9	6446.0	-1.6	-3.1	0	0
	nta-miR166h	UCGGACCAGGCUUCAUUCCCC	21	49450.2	17091.5	53404.9	6560.9	-1.5	-3.1	0	0
miR167	nta-miR167a	UGAAGCUGCCAGCAUGAUCUGG	22	1834.4	3497.5	3265.8	6321.6	0.9	0.9	5.75E-89	3.31E-59
	nta-miR167b	UGAAGCUGCCAGCAUGAUCUGG	22	1627.2	3731.1	3567.8	6264.4	1.2	0.8	3.5E-158	5.6E-24
	nta-miR167c	UGAAGCUGCCAGCAUGAUCUGG	22	1668.6	3341.0	3041.1	6094.9	1.0	1.0	7.2E-115	4.76E-74
	nta-miR167d	UGAAGCUGCCAGCAUGAUCUA	21	1478.6	3121.1	2458.1	5535.6	1.1	1.1	3.2E-131	7.05E-98
	nta-miR167e	UGAAGCUGCCAGCAUGAUCUA	21	1299.0	2850.1	2486.2	5684.8	1.1	1.1	3.4E-103	5.8E-97
miR168	nta-miR168a	UCGCUUGGUGCAGGUCGGGAC	21	7617.6	7429.8	8533.3	5086.3	0.0	-0.7	0.004713	9.6E-177
	nta-miR168b	UCGCUUGGUGCAGGUCGGGAC	21	7496.6	7604.1	8350.7	5057.3	0.0	-0.7	0.029621	4.4E-148
	nta-miR168c	UCGCUUGGUGCAGGUCGGGAC	21	7627.9	7466.5	8722.9	5021.9	0.0	-0.8	0.07815	1.4E-208
	nta-miR168d	UCGCUUGGUGCAGGUCGGGAA	21	3634.3	3265.7	2521.4	2569.4	-0.2	0.0	0.003428	0.02425
	nta-miR168e	UCGCUUGGUGCAGGUCGGGAA	21	3606.7	3336.5	2809.3	2553.1	-0.1	-0.1	0.060428	0.023894
miR169	nta-miR169a	CAGCCAAGGAUGACUUGCCGA	21	310.9	1703.0	526.7	3351.0	2.5	2.6	1.4E-212	4.5E-281
	nta-miR169b	CAGCCAAGGAUGACUUGCCGA	21	276.4	1745.5	421.4	3466.9	2.6	3.0	9.3E-211	0
	nta-miR169c	CAGCCAAGGAUGACUUGCCGA	21	314.4	1789.0	512.7	3251.8	2.5	2.6	6.6E-248	2.6E-287
	nta-miR169d	CAGCCAAGGAUGACUUGCCGA	21	310.9	1707.6	358.2	3349.6	2.5	3.2	6E-218	0
	nta-miR169e	CAGCCAAGGAUGACUUGCCGA	21	307.5	1841.2	484.6	3551.9	2.6	2.8	2.1E-249	0
	nta-miR169f	CAGCCAAGGAUGACUUGCCGA	21	324.7	1815.5	484.6	3474.5	2.5	2.8	4.6E-222	0
	nta-miR169g	CAGCCAAGGAUGACUUGCCGA	21	324.7	1690.4	393.3	3487.7	2.4	3.1	1.6E-198	0
	nta-miR169h	CAGCCAAGGAUGACUUGCCGA	21	266.0	1632.7	484.6	3471.7	2.6	2.8	3.4E-231	2.7E-307
	nta-miR169i	CAGCCAAGGAUGACUUGCCGA	21	300.6	1842.6	477.6	3163.3	2.6	2.7	4.3E-238	3.5E-308
	nta-miR169j	CAGCCAAGGAUGACUUGCCGA	21	245.3	1677.0	400.3	3424.6	2.8	3.0	6.4E-251	0
	nta-miR169k	CAGCCAAGGAUGACUUGCCGA	21	307.5	1524.8	484.6	3175.7	2.3	2.7	4.2E-167	8.7E-298
	nta-miR169l	CAGCCAAGGAUGACUUGCCGA	21	276.4	1845.9	484.6	3402.0	2.7	2.8	3.1E-270	0
	nta-miR169m	CAGCCAAGGAUGACUUGCCGA	21	355.8	1760.4	491.6	3180.8	2.3	2.6	4.1E-218	5E-298
	nta-miR169o	CAGCCAAGGAUGACUUGCCGA	21	304.0	1575.0	316.0	3380.0	2.4	3.4	3.7E-197	0
	nta-miR169p	CAGCCAAGGAUGACUUGCCGA	21	272.9	1820.8	449.5	3307.8	2.7	2.8	7.4E-259	0
	nta-miR169q	CAGCCAAGGAUGACUUGCCGG	21	673.7	2480.7	765.5	4346.5	1.9	2.5	3.6E-220	0
	nta-miR169r	CAGCCAAGGAUGACUUGCCGG	21	690.9	2549.2	779.6	3917.4	1.9	2.3	2E-216	8.2E-302
	nta-miR169s	CAGCCAAGGAUGACUUGCCGG	21	639.1	2726.2	807.7	4099.8	2.1	2.3	1.7E-292	0
	nta-miR169t	UAGCCAAGGAUGACUUGCCUU	21	925.9	2908.5	1826.0	4490.4	1.7	1.3	4.6E-214	6.6E-158
miR171	nta-miR171a	UAUUGGUGCGGUUCAAUGAGA	21	1858.6	4603.9	2542.4	4308.5	1.3	0.8	1.7E-249	7.7E-75
	nta-miR171b	UUGAGCCGCGCCAAUAUCACU	21	17632.7	59811.2	29062.3	105410.4	1.8	1.9	0	0
	nta-miR171c	UGAUUGAGCCGUGCCAAUAUC	21	2618.6	7008.6	4375.5	9618.8	1.4	1.1	0	5.7E-282
miR172	nta-miR172a	AGAAUCUUGAUGAUGCUGCAG	21	286.7	471.8	266.9	444.2	0.7	0.7	2.76E-11	4.47E-11
	nta-miR172b	AGAAUCAUGAUGAUGCUGCAU	21	297.1	267.1	175.6	276.9	-0.2	0.7	0.235287	2.38E-06
	nta-miR172c	AGAAUCUUGAUGAUGCUGCAU	21	145.1	109.1	98.3	178.8	-0.5	0.9	0.323111	9.76E-05
	nta-miR172d	AGAAUCUUGAUGAUGCUGCAU	21	134.7	109.6	70.2	128.5	-0.3	0.9	0.24627	0.000325
	nta-miR172e	AGAAUCUUGAUGAUGCUGCAU	21	96.7	159.8	56.2	98.4	0.7	0.8	0.050239	0.003424
	nta-miR172f	AGAAUCUUGAUGAUGCUGCAU	21	100.2	101.5	91.3	91.2	0.0	0.0	0.18307	0.123356
	nta-miR172g	AGAAUCUUGAUGAUGCUGCAU	21	117.5	109.3	98.3	144.1	-0.1	0.5	0.298106	0.443078
	nta-miR172h	AGAAUCUUGAUGAUGCUGCAU	21	107.1	126.0	28.1	120.9	0.2	2.1	0.286606	6.2E-13
	nta-miR172i	AGAAUCUUGAUGAUGCUGCAU	21	96.7	118.1	105.3	134.9	0.3	0.3	0.194279	0.258408
	nta-miR172j	GGAAUCUUGAUGAUGCUGCAU	21	224.6	281.4	252.8	284.4	0.3	0.2	0.315909	0.389459
miR1919	nta-miR1919	GAGCGAGUCAUCUGUGACAGG	21	5492.9	6021.9	2865.5	8580.2	0.1	1.6	0.03878	0
miR319	nta-miR319a	UUGGACUGAAGGGAGCUCCCU	21	7914.7	5074.6	17565.2	3011.8	-0.6	-2.5	8.9E-130	0
	nta-miR319b	UUGGACUGAAGGGAGCUCCCU	21	8246.3	4740.4	16919.0	2893.1	-0.8	-2.6	6.8E-209	0
miR390	nta-miR390a	AAGCUCAGGAGGGAUAGCACC	21	2984.8	3748.9	3048.1	3285.0	0.3	0.1	7.15E-18	0.052499
	nta-miR390b	AAGCUCAGGAGGGAUAGCGCC	21	2418.3	3518.5	1861.2	3809.6	0.5	1.0	2.78E-46	3E-103
	nta-miR390c	AAGCUCAGGAGGGAUAGCGCC	21	2207.5	3617.4	2092.9	4018.0	0.7	0.9	7.7E-69	8.45E-84
miR394	nta-miR394	UUGGCAUUCUGUCCACCUCC	20	5171.6	16375.7	3279.9	14625.2	1.7	2.1	0	0
miR395	nta-miR395a	CUGAAGUGUUUGGGGGAACUC	21	1109.0	1032.9	484.6	816.0	-0.1	0.7	0.492002	3.84E-15
	nta-miR395b	CUGAAGUGUUUGGGGGAACUC	21	984.6	1101.1	575.9	672.8	0.2	0.2	0.014945	0.041556
	nta-miR395c	CUGAAGUGUUUGGGGGAACUC	21	1070.9	970.4	470.6	717.3	-0.2	0.6	0.33554	1.56E-10
miR396	nta-miR396a	UUCCACAGCUUUCUUGAACUG	21	6356.6	4040.3	11174.0	2444.9	-0.7	-2.2	9.7E-97	0
	nta-miR396b	UUCCACAGCUUUCUUGAACUU	21	1178.0	955.9	2380.9	1020.0	-0.3	-1.2	5.04E-06	5.5E-107
	nta-miR396c	UUCCACAGCUUUCUUGAACUU	21	1223.0	962.4	2416.0	1057.6	-0.4	-1.2	0.078174	2E-99
miR397	nta-miR397	AUUGAGUGCAGCGUUGAUGU	20	1074.4	2443.7	842.8	3390.8	1.2	2.0	5.6E-115	1.8E-228
miR398	nta-miR398	UGUGUUCUCAGGUCGCCCCUG	21	7413.7	37620.5	6110.2	21892.4	2.3	1.8	0	0
miR399	nta-miR399a	CGCCAAAGGAGAGCUGCCCUG	21	2128.1	5878.8	3027.0	9332.7	1.5	1.6	0	0

	nta-miR399b	CGCCAAAGGAGAGCUGCCCUG	21	2283.5	5848.6	2626.7	9163.1	1.4	1.8	0	0
	nta-miR399c	CGCCAAAGGAGAGCUGCCCUG	21	2242.1	5853.2	3062.1	9166.1	1.4	1.6	0	0
	nta-miR399d	CGCCAAAGGAGAGCUGCCCUG	21	2207.5	5844.9	2837.4	9505.3	1.4	1.7	0	0
	nta-miR399e	CGCCAAAGGAGAGCUGCCCUG	21	2262.8	5870.3	2788.2	9524.0	1.4	1.8	0	0
	nta-miR399f	CGCCAAAGGAGAGCUGCCCUG	21	2397.5	5756.3	2739.1	8771.3	1.3	1.7	5.9E-285	0
	nta-miR399g	CGCCAAAGGAGAGCUGCCCUG	21	2117.7	6062.0	2767.2	9251.7	1.5	1.7	0	0
miR408	nta-miR408	UGCACUGCCUCUUCCCUGGCU	21	5175.1	14667.9	2591.6	7125.3	1.5	1.5	0	0
miR477	nta-miR477a	ACUCUCCCUCAAGGGCUUCUG	21	2874.3	4412.6	1826.0	4435.1	0.6	1.3	2.1E-69	1.4E-163
	nta-miR477b	UCUCUCCCUCAAGGGCUUCUC	21	1875.9	4657.4	1812.0	3512.2	1.3	0.9	2.6E-225	5.85E-74
miR479	nta-miR479a	CGUGAUAUUGGUUUGGCUCAUC	22	2076.3	2925.7	1868.2	2898.5	0.5	0.6	3.91E-25	6.05E-24
	nta-miR479b	CGUGAUAUUUGUUUGGCUCAUC	22	10039.3	15528.6	17003.3	47797.2	0.6	1.5	3.4E-157	0
miR482	nta-miR482a	UUUCCAAUUCCACCCAUUCCUA	22	801.5	1741.7	744.5	1505.3	1.1	1.0	5.25E-66	3.63E-38
	nta-miR482b-3p	UCUUGCCAAUGCCAUCCAUUCC	22	4739.8	18640.0	5906.6	16517.6	2.0	1.5	0	0
	nta-miR482b-5p	AGUGGGUGGAGUGGUAAGAUA	21	1945.0	3846.6	1397.6	5685.2	1.0	2.0	1.4E-134	0
	nta-miR482c	UUUCCAAUUCCACCCAUUCCUA	22	839.5	2476.3	962.2	2531.9	1.6	1.4	1.3E-165	2.9E-140
	nta-miR482d	UUCCCGACUCCCCCCAUACCAC	22	13818.7	53137.0	36640.4	95327.1	1.9	1.4	0	0
miR5303	nta-miR5303a	AAAAUGUGGCCGGAUACGUGU	21	4194.0	8398.0	2001.6	6309.2	1.0	1.6	1.2E-183	0
	nta-miR5303b	AAAAUGUGGCCGGAUACGUGU	21	4211.2	8476.4	1966.5	6493.5	1.0	1.7	8.5E-189	0
	nta-miR5303c	ACGGGUGCGGCUACAUUUUGG	21	17183.5	12890.8	4431.7	11421.8	-0.4	1.4	3.2E-137	0
miR6019	nta-miR6019a	UACAGGUGACUUGUAAAUGUUU	22	715.1	2459.1	1320.4	1770.4	1.8	0.4	6.5E-193	1.6E-10
	nta-miR6019b	UACAGGUGACUUGUAAAUGUUU	22	677.1	2155.5	1116.7	1426.0	1.7	0.4	7.3E-165	2.29E-09
miR6020	nta-miR6020a-3	AGAUACUCAGCAAAACAUUUAC	22	690.9	1142.5	597.0	1656.0	0.7	1.5	6.8E-14	4.4E-100
	nta-miR6020a-5	AAAUGUUUUUCGAGUAUCUUC	21	1706.6	529.3	273.9	621.5	-1.7	1.2	2.2E-131	4.23E-24
	nta-miR6020b	AAAUGUUCUUCGAGUAUCUUC	21	2860.5	1944.1	1215.0	2303.8	-0.6	0.9	2.03E-27	2.79E-54
miR6021	nta-miR6021	UUGGAAGAGGCUGCUAUUGGA	21	2812.1	2986.0	2008.7	5151.3	0.1	1.4	0.394606	2.4E-260
miR6024	nta-miR6024	UUUUAGCCAGAGUUGUUUUCCC	22	15048.6	24909.6	18653.8	30446.5	0.6	0.7	3.07E-11	0
miR6025	nta-miR6025a	UACCAACAAUUGAGAUAACAUC	22	894.8	3582.3	1524.0	2315.0	2.0	0.6	2.2E-298	2.47E-28
	nta-miR6025b	UGCCAACUAUUGAGAUGACAUC	22	666.8	2147.0	884.9	1614.2	1.7	0.9	2.8E-145	4.08E-47
	nta-miR6025c	UCAAUUGAGAUGACAUCUAGU	22	936.2	1358.6	1278.2	1125.0	0.5	-0.2	1.18E-15	0.184688
	nta-miR6025d	AACAAUUGAGAUAACAUCUAGG	23	473.3	393.7	723.4	325.2	-0.3	-1.2	0.016957	1.09E-25
	nta-miR6025e	UGCCAAUUAUAGAGAUGACAUC	23	739.3	2139.2	1179.9	1384.9	1.5	0.2	1.8E-153	0.018048
miR6144	nta-miR6144	UGGCAACUUCUUCAUCAUGCC	22	1053.7	2535.2	1201.0	2361.0	1.3	1.0	1E-121	2.13E-73
miR6145	nta-miR6145a	CAUUUUCACAUGUAGCACUGAC	23	1281.7	1639.7	884.9	1429.3	0.4	0.7	8.08E-11	5.25E-14
	nta-miR6145b	UUAUCAUACGUAGCACUAGCC	21	1278.2	1933.4	1467.9	1762.4	0.6	0.3	1.14E-24	3.9E-07
	nta-miR6145c	CAGUGCACAUAUAACAGUAA	20	207.3	368.6	358.2	565.5	0.8	0.6	5.05E-09	0.000361
	nta-miR6145d	AUUGUUACAUGUAACACUGGC	21	953.5	1187.1	934.1	2099.7	0.3	1.2	0.003488	2.24E-91
	nta-miR6145e	AUUGUUACAUGUAGCACUGGC	21	18468.7	11841.6	16954.2	5620.0	-0.6	-1.6	3.6E-303	0
	nta-miR6145f	AUCGUAACAUAUAGCACUAGC	21	321.3	512.3	463.5	653.7	0.7	0.5	9.8E-06	2.01E-06
miR6146	nta-miR6146a	UUUGUCCAAUGAAACACUUAUC	23	1026.0	839.5	1706.7	734.4	-0.3	-1.2	0.000844	5.4E-73
	nta-miR6146b	UUUGUCCAAUGAAAUACUUAUC	23	2656.6	1137.3	1503.0	624.5	-1.2	-1.3	1.3E-131	7.95E-69
miR6147	nta-miR6147	UGACAUCUUCAAAACCCACUA	22	563.1	1332.7	765.5	1217.6	1.2	0.7	1.12E-56	1.24E-10
miR6148	nta-miR6148a	UACGUCGAUCGAUUGUUCUUA	22	829.1	1896.3	653.2	2040.5	1.2	1.6	1.63E-89	5.5E-145
	nta-miR6148b	UGUGUUAAUCGUUUGUUCUCA	22	1219.5	1499.4	1552.1	1199.6	0.3	-0.4	1.51E-05	1.05E-05
miR6149	nta-miR6149a	UUGAUACGCACCUGAAUCGGC	22	23011.6	8535.6	24061.7	5060.5	-1.4	-2.3	0	0
	nta-miR6149b	UUGAUACGCACCUGAAUCGGC	22	22994.3	8633.4	23563.0	5245.0	-1.4	-2.2	0	0
miR6150	nta-miR6150	AGAUUUGUUUGAUCGUCUUGGC	23	1482.1	1841.2	1334.4	2153.0	0.3	0.7	2.52E-07	1.4E-27
miR6151	nta-miR6151a	UGAAUGUGAGGCAUUGGAUUGA	23	269.5	450.8	203.7	405.3	0.7	1.0	1.27E-05	8.75E-15
	nta-miR6151b	UGAAUGUGAGGCAUUGGAUUGA	23	321.3	387.3	196.7	462.8	0.3	1.2	0.333788	1.24E-23
	nta-miR6151c	UGAAUGUGAGGCAUUGGAUUGA	23	286.7	410.6	140.5	461.1	0.5	1.7	0.006506	3.73E-38
	nta-miR6151d	UGAAUGUGAGGCAUUGGAUUGA	23	362.7	436.3	168.6	441.6	0.2	1.4	0.152726	2.66E-28
	nta-miR6151e	UGAAUGUGAGGCAUUGGAUUGA	23	314.4	341.8	203.7	475.8	0.1	1.2	0.346097	1.15E-20
	nta-miR6151f	UGAGUGUGAGGCAUUGGAUUGA	23	490.6	533.6	316.0	756.4	0.1	1.3	0.346033	6.71E-41
	nta-miR6151g	UGAGUGUGAGGCGUUGGAUUGA	23	2428.6	1479.0	2380.9	1595.1	-0.7	-0.6	6.15E-50	1.13E-29
	nta-miR6151h	UGAGUGUGAGGCGUUGGAUUGA	23	2566.8	1330.3	2563.5	1575.2	-0.9	-0.7	1.46E-78	2.3E-39
	nta-miR6151i	UGAGUGUGAGGCGUUGGAUUGA	23	2376.8	1453.1	2640.7	1732.3	-0.7	-0.6	3.67E-46	5.05E-29
miR6152	nta-miR6152a	UAUUGUAUUCGACUGUAUUCAC	24	535.5	789.1	590.0	1053.3	0.6	0.8	3.52E-10	2.89E-13
	nta-miR6152b	UAUUGUAUUCGACUGUAUUCAC	24	507.8	746.5	618.0	1022.8	0.5	0.7	0.000218	1.65E-15
miR6153	nta-miR6153	UAGGACCAUAUUCACUAUUUG	21	2725.7	2833.3	5127.0	3972.7	0.1	-0.4	0.345726	2.28E-24
miR6154	nta-miR6154a	UGGGUCUCCUGGAGAAAGGUC	21	2097.0	4601.7	1889.3	3961.7	1.1	1.1	2.3E-175	5.8E-144
	nta-miR6154b	UGGGUCUCCUGGAGAAAGGUC	21	2114.3	4479.7	1875.2	3939.0	1.1	1.1	4.1E-170	4.8E-120
miR6155	nta-miR6155	UAAGGUUGCCUUGCUCUUGCA	21	2266.3	4207.9	1790.9	4090.0	0.9	1.2	1.6E-111	1.1E-177
miR6156	nta-miR6156	UUGAAGAUGUUCUAUUUCUGU	21	224.6	447.3	238.8	440.3	1.0	0.9	2.57E-15	1.75E-11
miR6157	nta-miR6157	UGGUAGACGUAGGAUUUGAAGA	22	2007.2	2596.5	1566.2	2183.5	0.4	0.4	5.45E-11	0.004925
miR6158	nta-miR6158a	AAGUUCGAUUUGUACGAAGGGC	22	1419.9	3813.9	1425.7	3911.6	1.4	1.4	7.7E-235	1.9E-198
	nta-miR6158b	AAGUUCGAUUUGUACGAAGGGC	22	1402.6	3871.7	1474.9	3827.0	1.5	1.4	8.7E-249	2E-185
	nta-miR6158c	AAGUUCGAUUUGUACGAAGGGC	22	1433.7	3949.0	1503.0	3964.5	1.5	1.4	8.7E-242	3.3E-172
miR6159	nta-miR6159	UAGCAUAGAAUUCUCGCACCUA	22	3043.6	4947.8	4466.8	3393.1	0.7	-0.4	5.75E-87	1.32E-30
miR6160	nta-miR6160	GCAUAUAUGGGCCAACUGUGUA	24	3675.8	7230.8	2605.6	5028.3	1.0	0.9	4.7E-243	6.2E-123
miR6161	nta-miR6161a	GCUGGACCGGUAUACUUUGCUG	24	3672.3	4980.4	2626.7	5502.0	0.4	1.1	2.43E-44	1.2E-216
	nta-miR6161b	GCUGGACCGGUAUACUUUGCUG	24	3503.0	4964.9	2514.3	5309.3	0.5	1.1	1.3E-49	2.9E-195
	nta-miR6161c	AAUAUACUGGAGUUCGGUGCAC	24	6494.8	9666.0	4228.0	8858.7	0.6	1.1	3.9E-133	0
	nta-miR6161d	UGAACUCCAGCAUAUUAUACU	21	863.7	2632.0	1812.0	2213.5	1.6	0.3	1.3E-196	0.341485
miR6162	nta-miR6162	AGAAAAAUGGUAGCCAUUGGA	21	2577.2	3478.9	1826.0	3205.7	0.4	0.8	1.53E-25	2.36E-65
miR6163	nta-miR6163	UGGAAGUACUGCCUAAGUUUGA	22	1793.0	2509.2	2689.9	2608.9	0.5	0.0	9E-12	0.320612
miR6164	nta-miR6164a	UCACAUAAAUUGAAACGGAGG	21	407.7	851.5	435.4	768.4	1.1	0.8	4.2E-31	9.75E-12
	nta-miR6164b	UCACAUAAAUUGAAACGGAGG	21	414.6	762.8	393.3	848.4	0.9	1.1	6.51E-24	1.64E-33
miR827	nta-miR827	UUAGAUGAACAUCAACAAACA	21	545.8	531.6	428.4	781.2	0.0	0.9	0.531752	2.72E-16

[a]TPM value indicates the expression level of miRNA; TPM value = counts of this miRNA/ total counts of this sample×1000000.

[b]Fold change (Cd vs Control) is $\log_2 N$, $\log_2 N \geqslant 1.5$ are up-regulated and $0 < |\log_2 N| < 1.5$ are unchanged and $\log_2 N \leqslant -1.5$ are down-regulated, $p < 0.01$.

G–Cd, G+Cd, Y-Cd and Y+Cd correspond to hydroponic tobacco Guiyan 1 grown in basic nutrition solution (BNS), Guiyan 1 in BNS+ 50μM Cd, Yunyan 2 in BNS and Yunyan 2 in BNS + 50μM Cd, respectively.

Table S3 Novel miRNAs expressed in both tobacco genotypes Guiyan1 (G) and Yunyan2 (Y) under control and 50 μM Cd conditions at day 5.

miRNA name	Chromosome	Start	End	Orientati	Abundance	Sequence	**Length (nt**	Genomic h	Hairpin l	Hairpin G	Minimum f	Adjusted	Randfold	miRNA*	TPM[a]				Fold change[b]	
															G-Cd	G+Cd	Y-Cd	Y+Cd	G	Y
novel-miR1	Niben044Scf00002137_350188	32708	32728	+	403	ATCATGCTATCCCTTTGGACT	21	2	126	40.5	−54.6	−43.3	0.009901	TCCAAAGGG	122.8	123.2	129.5	130.5	1.0	1.0
novel-miR2	Niben044Scf00014823_335993	245585	245605	+	246	GGAATGTTGTCTGGCTCGAGG	21	1	209	34.9	−74.6	−35.7	0.009901	NO	37.5	37.5	41.4	39.6	1.0	0.0
novel-miR3	Niben044Scf00004045_395210	262611	262633	+	16	CTAGAACTCCAGCATAATATACT	23	7	87	35.6	−28.2	−32.4	0.009901	NO	9.8	10.1	10.4	9.8	1.0	−0.1
novel-miR4	Niben044Scf00004103_360652	102876	102897	+	15	TCCACATCCTTGTTGATAACTG	22	10	89	43.8	−29.6	−33.3	0.009901	NO	9.1	8.8	9.7	9.1	0.0	−0.1
novel-miR5	Niben044Scf00023006_384368	116423	116443	+	41	TTGTGAGACAAAAAGAAGCCT	21	1	152	38.2	−87.4	−57.5	0.009901	NO	6.2	6.4	6.6	6.5	1.0	0.0
novel-miR6	Niben044Scf00002137_350188	32634	32654	+	18	TCCAAAGGGATCGCATTGATC	21	2	117	40.2	−52.1	−44.5	0.009901	GATCATGCT	5.5	5.8	5.5	5.7	0.9	1.0
novel-miR7	Niben044Scf00012994_341128	316112	316134	−	28	ATTTGGTCTAGTGGTATGATTCT	23	2	86	37.2	−21.7	−25.2	0.009901	NO	4.3	4.4	4.7	4.6	1.0	0.0
novel-miR8	Niben044Scf00001682_380483	208795	208815	−	13	TTGGTGATATTTCTTCGGATT	21	4	210	30.5	−122.1	−58.1	0.009901	NO	4.0	5.2	4.1	5.4	0.8	0.8
novel-miR9	Niben044Scf00006151_355967	99678	99698	+	10	TTCTTTTGGACAAGTAGCACC	21	2	233	34.8	−139.4	−59.8	0.009901	NO	3.0	3.1	3.1	3.1	1.0	1.0
novel-miR10	Niben044Scf00017997_399588	109107	109127	+	15	CATAGCCAATCTTTGGAGCCT	21	1	313	31.0	−162.4	−51.9	0.009901	TCAAGGTTC	2.3	2.3	2.4	2.4	1.0	0.0
novel-miR11	Niben044Scf00002443_315754	205534	205556	−	10	TATCGGTTTAGCTCTTATCGGGC	23	1	112	45.5	−34.7	−31.0	0.049505	NO	1.5	1.5	1.6	1.7	1.0	0.9
novel-miR12	Niben044Scf00009074_604417	219730	219751	−	9	CTGGGTGGTGTAGTCGGTTATC	22	1	155	41.3	−42.0	−27.1	0.029703	NO	1.4	1.4	1.6	1.6	1.0	1.0
novel-miR13	Niben044Scf00006338_477699	443234	443256	−	8	TTAACTTTTGAACTTGGAACTCA	23	8	208	33.2	−85.0	−40.9	0.009901	NO	1.2	1.3	1.4	1.2	0.9	−0.1
novel-miR14	Niben044Scf00002388_322526	310689	310711	−	8	GATACATGTGTCGCAGAAGACTT	23	2	139	30.9	−41.5	−29.9	0.009901	NO	1.2	1.3	1.4	1.3	0.9	−0.1
novel-miR15	Niben044Scf00003359_572295	508425	508446	−	7	TGAAGAAGAATGAACTAGCACC	22	6	55	45.5	−17.0	−30.9	0.019802	NO	1.1	1.1	1.2	1.2	1.0	1.0
novel-miR16	Niben044Scf00002583_361192	317931	317951	+	6	AATCCGAGCCCCACATTCATC	21	1	97	45.4	−50.3	−51.9	0.009901	NO	0.9	1.0	0.9	0.9	0.9	1.0
novel-miR17	Niben044Scf00003611_445722	318844	318865	+	21	CGTCTCCTTGATTGTGGTAGGC	22	2	95	60.0	−38.9	−40.9	0.009901	NO	3.2	3.4	0.0	3.3	—	↑
novel-miR18	Niben044Scf00004802_433360	238081	238103	−	12	AAGGATTCAAGGTAGAGCTGCTT	23	1	73	43.8	−18.7	−25.6	0.09901	NO	1.8	1.9	0.0	1.1	—	↑
novel-miR19	Niben044Scf00000377_326229	90708	90729	+	5	TATTTGTAGGAAAAGGTTACCT	22	3	50	40.0	−16.1	−32.2	0.029703	NO	1.5	0.0	1.7	0.0	↓	↓
novel-miR20	Niben044Scf00002190_346493	112848	112870	−	10	CAGGATAAGTATGTTGGAACTCC	23	3	142	35.9	−54.8	−38.6	0.009901	NO	1.5	0.7	0.0	0.0	—	—
novel-miR21	Niben044Scf00019316_317706	167182	167202	+	5	ACGAGGTTCGGACAAGTTGCA	21	5	111	45.9	−34.3	−30.9	0.009901	NO	0.8	0.8	0.0	0.4	—	↑
novel-miR22	Niben044Scf00003134_352543	336479	336500	−	5	CAACATGTGGAAGATCTTAGCA	22	5	155	43.9	−47.2	−30.5	0.009901	NO	0.8	0.8	0.0	0.9	—	↑
novel-miR23	Niben044Scf00016243_362612	196130	196151	+	5	AACCAAGGATATGTAGGCAGCT	22	4	58	43.1	−14.7	−25.3	0.089109	NO	0.8	0.0	0.0	0.0	↓	—
novel-miR24	Niben044Scf00022866_467875	216492	216511	−	106	TCCAGCGGCTGGAAGAGCAC	20	1	153	56.2	−52.4	−34.2	0.089109	NO	0.0	0.0	18.3	8.7	—	—
novel-miR25	Niben044Scf00004550_314476	73035	73056	−	7	TATTATGCTGGACCGGTATACT	22	12	63	36.5	−22.1	−35.1	0.01	NO	0.0	0.0	3.6	0.0	—	↓
novel-miR26	Niben044Scf00009193_366732	292363	292384	+	18	TGAGTGTGAGGCGTTGGATTGA	22	1	199	41.7	−78.9	−39.7	0.009901	AATCCAAGC	0.0	2.8	3.1	3.0	↑	—
novel-miR27	Niben044Scf00003611_445722	318843	318864	+	18	CCGTCTCCTTGATTGTGGTAGG	22	2	124	62.1	−51.8	−41.8	0.089109	NO	0.0	0.0	3.1	0.0	—	↓
novel-miR28	Niben044Scf00012719_341801	236835	236857	+	5	ATAATATACTGGAGATTGGAGCC	23	1	159	39.0	−54.6	−34.3	0.009901	NO	0.0	0.0	0.9	0.4	—	—
novel-miR29	Niben044Scf00013296_316665	20878	20900	+	79	AGAGAGACTGTTTCCGATAGACC	23	4	283	49.1	−184.1	−65.1	0.009901	NO	0.0	0.0	0.0	7.0	—	↑
novel-miR30	Niben044Scf00003611_445722	317845	317866	+	149	GTGAGCATACCTGTCGGGACCC	22	2	96	55.2	−29.0	−30.2	0.089109	TGGTGGAGG	0.0	0.0	0.0	12.7	—	↑

[a]TPM value indicates the expression level of miRNA; TPM value = counts of this miRNA/ total counts of this sample×1000000.

[b]Fold change (Cd vs Control) is $\log_2 N$, $\log_2 N \geqslant 1.5$ are up-regulated and $0 < |\log_2 N| < 1.5$ are unchanged and $\log_2 N \leqslant -1.5$ are down-regulated, $p < 0.01$.

"↑" indicates only expressed in Cd-treated, "↓" only expressed in control, " - " not expressed in both conditions or unchanged.

G–Cd, G+Cd, Y-Cd and Y+Cd correspond to hydroponic tobacco Guiyan 1 grown in basic nutrition solution (BNS), Guiyan 1 in BNS+ 50μM Cd, Yunyan 2 in BNS and Yunyan 2 in BNS + 50μM Cd, respectively.

Table S4 Known miRNAs up-regulated in both genotypes Guiyan1 (G) and Yunyan2 (Y) under 50 μM Cd stress.

Family name	miRNA name	Sequence	Length (nt)	TPM[a]				Fold change[b]		Target gene	Annotation
				G-Cd	G+Cd	Y-Cd	Y+Cd	G	Y		
miR169	nta-miR169a	CAGCCAAGGAUGACUUGCCGA	21	310.9	1703.0	526.7	3351.0	2.5	2.6	TC164425, TC	Cytochrome P450 like_TBP (N. tabacum), Salicylic acid-binding p
	nta-miR169b	CAGCCAAGGAUGACUUGCCGA	21	276.4	1745.5	421.4	3466.9	2.6	3.0		
	nta-miR169c	CAGCCAAGGAUGACUUGCCGA	21	314.4	1789.0	512.7	3251.8	2.5	2.6		
	nta-miR169d	CAGCCAAGGAUGACUUGCCGA	21	310.9	1707.6	358.2	3349.6	2.5	3.2		
	nta-miR169e	CAGCCAAGGAUGACUUGCCGA	21	307.5	1841.2	484.6	3551.9	2.6	2.8		
	nta-miR169f	CAGCCAAGGAUGACUUGCCGA	21	324.7	1815.5	484.6	3474.5	2.5	2.8		
	nta-miR169g	CAGCCAAGGAUGACUUGCCGA	21	324.7	1690.4	393.3	3487.7	2.4	3.1		
	nta-miR169h	CAGCCAAGGAUGACUUGCCGA	21	266.0	1632.7	484.6	3471.7	2.6	2.8		
	nta-miR169i	CAGCCAAGGAUGACUUGCCGA	21	300.6	1842.6	477.6	3163.3	2.6	2.7		
	nta-miR169j	CAGCCAAGGAUGACUUGCCGA	21	245.3	1677.0	400.3	3424.6	2.8	3.0		
	nta-miR169k	CAGCCAAGGAUGACUUGCCGA	21	307.5	1524.8	484.6	3175.7	2.3	2.7		
	nta-miR169l	CAGCCAAGGAUGACUUGCCGA	21	276.4	1845.9	484.6	3402.0	2.7	2.8		
	nta-miR169m	CAGCCAAGGAUGACUUGCCGA	21	355.8	1760.4	491.6	3180.8	2.3	2.6		
	nta-miR169o	CAGCCAAGGAUGACUUGCCGA	21	304.0	1575.0	316.0	3380.0	2.4	3.4		
	nta-miR169p	CAGCCAAGGAUGACUUGCCGA	21	272.9	1820.8	449.5	3307.8	2.7	2.8		
	nta-miR169q	CAGCCAAGGAUGACUUGCCGG	21	673.7	2480.7	765.5	4346.5	1.9	2.5		
	nta-miR169r	CAGCCAAGGAUGACUUGCCGG	21	690.9	2549.2	779.6	3917.4	1.9	2.3		
	nta-miR169s	CAGCCAAGGAUGACUUGCCGG	21	639.1	2726.2	807.7	4099.8	2.1	2.3		
miR171	nta-miR171b	UUGAGCCGCGCCAAUAUCACU	21	17632.7	59811.2	29062.3	105410.4	1.8	1.9	TC134811	Hairy meristem (*Petunia x hybrida*)
miR394	nta-miR394	UUGGCAUUCUGUCCACCUCC	20	5171.6	16375.7	3279.9	14625.2	1.7	2.1	TC122940	Nitrilase (*N. tabacum*)
										TC141337	
										EB433013	
										FS429526	
miR398	nta-miR398	UGUGUUCUCAGGUCGCCCCUG	21	7413.7	37620.5	6110.2	21892.4	2.3	1.8	TC146710	Metallothionein-like protein type 2 (*N. tabacum*)
										TC153262	
										TC168311	
										CV020687	
										EB449403	
										EB448414	
miR399	nta-miR399a	CGCCAAAGGAGAGCUGCCCUG	21	2128.1	5878.8	3027.0	9332.7	1.5	1.6	AM828792 AM8	Inorganic phosphate transporter 1-7/AtPht1;7 (*A. thaliana*)
	nta-miR399g	CGCCAAAGGAGAGCUGCCCUG	21	2117.7	6062.0	2767.2	9251.7	1.5	1.7		
miR408	nta-miR408	UGCACUGCCUCUUCCCUGGCU	21	5175.1	14667.9	2591.6	7125.3	1.5	1.5	TC122858	Heat shock protein (*N. tabacum*)
										TC141347	
										TC135872	
										HO059626	
										HS085296	
										TC125195	
										TC126000	
										FG133364	
										FG137124	
miR482	nta-miR482b-3	UCUUGCCAAUGCCAUCCAUUC	22	4739.8	18640.0	5906.6	16517.6	2.0	1.5	TC123107	Glucosyltransferase (*N. tabacum*)
										TC158882	

[a]TPM value indicates the expression level of miRNA; TPM value = counts of this miRNA/ total counts of this sample×1000000.

[b]Fold change (Cd vs Control) is $\log_2 N$, $\log_2 N \geqslant 1.5$ are up-regulated and $0 < |\log_2 N| < 1.5$ are unchanged and $\log_2 N \leqslant -1.5$ are down-regulated, $p < 0.01$.

G–Cd, G+Cd, Y-Cd and Y+Cd correspond to hydroponic tobacco Guiyan 1 grown in basic nutrition solution (BNS), Guiyan 1 in BNS+ 50μM Cd, Yunyan 2 in BNS and Yunyan 2 in BNS + 50μM Cd, respectively.

Table S5 Targets prediction of known miRNAs.

Family name	miRNA name	Target gene	Gene length (nt)	Tot score	Tot energ	Max score	Max energ	Positions	Description
miR1446	nta-miR1446	TC125184	905	495	-71.7	172	-26.1	341 113 793	Ubiquitin (*S. lycopersicum*)
miR156	nta-miR156a/b/c/d/e/f/g/h/i/	TC152836	640	182	-35.3	182	-35.3	405	Squamosa promoter-binding-like protein 12 (*O. sativa*)
miR159	nta-miR159	TC148124	1696	179	-30.7	179	-30.7	431	TGA10 transcription factor (*N. tabacum*)
		TC154362	664	179	-30.7	179	-30.7	521	
		EB446223	878	179	-30.7	179	-30.7	462	
		FG172155	796	163	-20.7	163	-20.7	697	
miR160	nta-miR160a/b/c/d	EH663826	1004	300	-43.8	160	-23.7	189 759	Kinesin-like protein NACK1 (*N. tabacum*)
		TC125766	3208	160	-23.7	160	-23.7	916	
miR162	nta-miR162a/b	TC169250	715	182	-29.1	182	-29.1	260	Auxin-regulated protein-like (*A. thaliana*)
miR164	nta-miR164a/b/c	TC156300	719	187	-35.8	187	-35.8	238	NAC5 protein (*Glycine max*)
miR166	nta-miR166a/b/c/d/e/f/g/h	TC126889	1192	191	-40.6	191	-40.6	348	HD-ZIPIII protein (*N. sylvestris*)
miR167	nta-miR167a/b/c	TC131622	1731	319	-44.9	166	-23.6	384 1132	Villin 2 (*N. tabacum*)
	nta-miR167d/e	FG138379	849	335	-44.8	171	-24.4	343 180	Chromosome chr13 scaffold_17 (*V. vinifera*)
		FG144930	876	335	-44.8	171	-24.4	354 106	
miR168	nta-miR168a/b/c/d/e	TC126364	878	169	-30.2	169	-30.2	347	CaMB-channel protein (*N. tabacum*)
		BP532584	431	169	-30.2	169	-30.2	52	
miR169	nta-miR169a/b/c/d/e/f/g/h/i/	TC164425	1619	301	-44.9	153	-24.3	1327 832	Cytochrome P450 like TBP (*N. tabacum*)
		TC123257	1271	295	-40.3	148	-20.3	549 704	Salicylic acid-binding protein 2 (*N. tabacum*)
		TC128216	1040	183	-34.4	183	-34.4	561	CCAAT-binding transcription factor subunit B (*N. tabacum*)
		TC143413	1544	183	-34.4	183	-34.4	1190	
		TC167045	949	183	-34.4	183	-34.4	562	
		NP917237	966	183	-34.4	183	-34.4	577	
	nta-miR169t	TC128216	1040	183	-34.4	183	-34.4	561	CCAAT-binding transcription factor subunit B (*N. tabacum*)
		TC143413	1544	183	-34.4	183	-34.4	1190	
		TC167045	949	183	-34.4	183	-34.4	562	
		NP917237	966	183	-34.4	183	-34.4	577	
miR171	nta-miR171a	TC129266	1598	334	-41.9	167	-21.0	506 694	Chromosome chr11 scaffold_13 (*V. vinifera*)
	nta-miR171b	TC134811	1014	195	-38.4	195	-38.4	407	Hairy meristem (*Petunia x hybrida*)
	nta-miR171c	TC127385	1636	191	-39.5	191	-39.5	284	Chromosome chr15 scaffold_37 (*V. vinifera*)
		TC134811	1014	191	-39.5	191	-39.5	410	Hairy meristem (*Petunia x hybrida*)
miR172	nta-miR172a/c/d/e/f/g/h/i/j	TC146861	813	182	-25.7	182	-25.7	277	MYB transcription factor (*P. latifolia*)
		FS412876	575	182	-25.7	182	-25.7	276	MYB transcription factor MYB149 (*Glycine max*)
	nta-miR172b	AM792182	379	179	-23.1	179	-23.1	201	TINY-like protein (*P. trichocarpa*)
miR1919	nta-miR1919	TC123361	1811	169	-22.9	169	-22.9	1353	Glucose-1-phosphate adenylyltransferase (*N. tabacum*)
miR319	nta-miR319a/b	TC150601	966	175	-36.1	175	-36.1	367	SlTCP3 (*S. lycopersicu*m)
miR390	nta-miR390a	TC157664	1610	318	-47.0	161	-24.8	1315 1170	CC-NBS-LRR protein (*S. tuberosum*)
	nta-miR390b/c	TC157664	1610	318	-47.0	161	-24.8	1315 1170	CC-NBS-LRR protein (*S. tuberosum*)
		TC122853	3924	308	-41.2	154	-20.6	3835 3847	DNA-directed RNA polymerase 1B (*N. tabacum*)
miR394	nta-miR394	TC122940	1316	170	-23.0	170	-23.0	584	Nitrilase (*N. tabacum*)
		TC141337	906	170	-23.0	170	-23.0	712	
		EB433013	680	170	-23.0	170	-23.0	318	
		FS429526	527	170	-23.0	170	-23.0	406	
miR395	nta-miR395a/b/c	FS411240	618	325	-42.4	164	-21.6	100 571	Xanthine/uracil permease family protein (*S. lycopersicum*)
miR396	nta-miR396a/b/c	TC131399	5645	317	-44.0	162	-22.0	3777 5210	Growth-regulating factors (*N. tabacum*)
		AM814093	509	161	-20.0	161	-20.0	266	
		TC144468	739	155	-22.0	155	-22.0	306	
miR397	nta-miR397	TC137837	1984	190	-34.2	190	-34.2	746	Laccase/Diphenol oxidase (*N. tabacum*)
miR398	nta-miR398	TC146710	541	162	-21.3	162	-21.3	148	Metallothionein-like protein type 2 (*N. tabacum*)
		TC153262	1013	162	-21.3	162	-21.3	620	
		TC168311	808	162	-21.3	162	-21.3	127	
		CV020687	348	162	-21.3	162	-21.3	127	
		EB449403	715	162	-21.3	162	-21.3	576	
		EB448414	787	162	-21.3	162	-21.3	247	
miR399	nta-miR399a/b/c/d/e/f/g	AM828792	400	183	-33.3	183	-33.3	112	Inorganic phosphate transporter 1-7/AtPht1;7 (*A. thaliana*)
		AM816750	571	183	-33.3	183	-33.3	315	
miR408	nta-miR408	TC122858	2265	299	-44.0	154	-24.0	2000 1228	Heat shock protein (*N. tabacum*)
		TC141347	2408	164	-25.7	164	-25.7	2141	
		TC135872	842	154	-24.0	154	-24.0	697	
		HO059626	626	146	-22.5	146	-22.5	457	
		HS085296	664	146	-22.5	146	-22.5	584	
		TC125195	931	145	-20.0	145	-20.0	643	
		TC126000	904	145	-20.0	145	-20.0	350	
		FG133364	799	145	-20.0	145	-20.0	484	
		FG137124	736	145	-20.0	145	-20.0	410	
miR477	nta-miR477a/b	TC140202	835	335	-54.4	173	-29.4	474 567	Sucrose-6-phosphate synthase A (*N. tabacum*)
		TC141556	3776	335	-54.4	173	-29.4	513 606	
miR479	nta-miR479a	AM835468	568	176	-20.5	176	-20.5	416	Gibberellin 2-oxidase 3 (*N. tabacum*)
		TC139727	1344	176	-24.1	176	-24.1	357	Per1-like family protein (*S. tuberosum*)
		FS429339	529	176	-24.1	176	-24.1	446	
	nta-miR479b	TC139727	1344	168	-21.3	168	-21.3	357	Per1-like family protein (*S. tuberosum*)
		FS429339	529	168	-21.3	168	-21.3	446	
miR482	nta-miR482a/c	TC125874	1271	177	-29.5	177	-29.5	349	Glucose-6-phosphate isomerase (*S. lycopersicum*)
		TC131822	873	177	-29.5	177	-29.5	451	
	nta-miR482b-3p	TC123107	1662	176	-22.8	176	-22.8	1466	Glucosyltransferase (*N. tabacum*)
		TC158882	533	176	-22.8	176	-22.8	213	
	nta-miR482b-5p	TC127715	959	312	-49.0	159	-25.5	242 286	Proline-rich protein (*S. lycopersicum*)
	nta-miR482d	TC168260	622	172	-25.3	172	-25.3	179	Cyclic nucleotide-gated calmodulin-binding ion channel (*N. tab*
miR5303	nta-miR5303a/b	BP133050	548	167	-22.2	167	-22.2	350	Cytosolic ascorbate peroxidase (*N. tabacum*)
	nta-miR5303c	TC159100	526	445	-68.4	155	-24.1	342 190 160	TA20 protein (*N. tabacum*)
miR6019	nta-miR6019a/b	FS424154	570	196	-35.8	196	-35.8	179	Avr9/Cf-9 rapidly elicited protein 4 (*N. tabacum*)
miR6020	nta-miR6020a-3p	TC126565	881	178	-24.3	178	-24.3	160	Steroid membrane binding protein-like (*O. sativa*)
	nta-miR6020a-5p	TC142703	2834	191	-30.6	191	-30.6	175	Avr9/Cf-9 rapidly elicited protein 4 (*N. tabacum*)
		FS424154	570	191	-30.6	191	-30.6	166	
	nta-miR6020b	TC138322	931	162	-20.0	162	-20.0	271	Transformer-SR ribonucleoprotein (*N. tabacum*)
		TC140062	946	162	-20.0	162	-20.0	236	
		CV017868	539	162	-20.0	162	-20.0	361	
miR6021	nta-miR6021	FG137159	880	324	-42.2	166	-22.1	372 292	Copper P1B-ATPase (*Glycine max*)
miR6024	nta-miR6024	TC124430	885	175	-27.9	175	-27.9	262	Serine acetyltransferase (*N. plumbaginifolia*)
		TC126011	979	175	-27.9	175	-27.9	235	
		TC126255	1396	175	-27.9	175	-27.9	164	
miR6025	nta-miR6025a	TC135300	853	179	-22.6	179	-22.6	806	Ubiquitin carrier protein (*N. tabacum*)
	nta-miR6025b	TC124063	2581	180	-22.2	180	-22.2	2533	NTH23 protein (*N. tabacum*)
	nta-miR6025c	TC123965	1959	174	-22.4	174	-22.4	550	Vacuolar H+-ATPase B subunit (*N. tabacum*)
		TC140906	1468	174	-22.4	174	-22.4	519	
		TC164662	1447	174	-22.4	174	-22.4	562	
		TC156686	667	170	-23.8	170	-23.8	610	
	nta-miR6025d	TC141163	1137	192	-27.4	192	-27.4	710	NBS-LRR type resistance protein (*O. sativa*)
	nta-miR6025e	TC123463	2585	174	-23.3	174	-23.3	2392	N-ethylmaleimide sensitive fusion protein (*N. tabacum*)
miR6144	nta-miR6144	TC134426	677	181	-29.5	181	-29.5	252	Histone H3.2 (*A. thaliana*)
miR6145	nta-miR6145a	TC129271	1002	183	-23.8	183	-23.8	170	RNA Binding Protein 45 (*N. plumbaginifolia*)
	nta-miR6145b	TC137902	913	167	-22.0	167	-22.0	368	DnaJ-like protein isoform (*S. phureja*)
		FG638744	646	167	-22.0	167	-22.0	252	
	nta-miR6145c	TC127308	1385	164	-21.4	164	-21.4	1111	NADPH-cytochrome P450 oxidoreductase (*N. tabacum*)
		TC131140	1383	164	-21.4	164	-21.4	412	

		TC133142	901	164	-21.4	164	-21.4	539	
	nta-miR6145d	TC159048	737	175	-23.2	175	-23.2	408	Basic form of pathogenesis-related protein 1 precursor (*N. tab*
	nta-miR6145e	TC125028	2118	171	-22.2	171	-22.2	1496	Ferrochelatase (*N. tabacum*)
		TC140088	970	171	-22.2	171	-22.2	203	
		FG637935	765	171	-22.2	171	-22.2	677	
	nta-miR6145f	NP13074235	3631	176	-25.7	176	-25.7	3147	N-like protein (*N. tabacum*)
miR6146	nta-miR6146a/b	TC136742	1735	176	-23.1	176	-23.1	1524	CYP72 (*N. tabacum*)
		TC148572	719	176	-23.1	176	-23.1	335	
miR6147	nta-miR6147	TC135051	1607	172	-20.7	172	-20.7	1205	Glutamate dehydrogenase B (*. plumbaginifolia*)
		TC158298	807	172	-20.7	172	-20.7	465	
miR6148	nta-miR6148a	TC142950	842	332	-40.4	166	-20.2	712 772	ACC oxidase ACO3 (*N. attenuata*)
	nta-miR6148b	TC160770	564	179	-20.2	179	-20.2	40	Chromosome chr11 scaffold_13 (*V. vinifera*)
miR6149	nta-miR6149a/b	FS390832	362	165	-22.9	165	-22.9	319	TCTR2 protein (*S. lycopersicum*)
miR6150	nta-miR6150	TC165730	1028	179	-21.5	179	-21.5	868	Retrotransposon gag protein (*S. demissum*)
miR6151	nta-miR6151a/b/c/d/e	TC123188	947	180	-24.0	180	-24.0	560	*Catalase isozyme 1 (N. tabacum*)
		TC124634	908	180	-24.0	180	-24.0	490	
		TC124875	865	180	-24.0	180	-24.0	421	
		TC162875	1935	180	-24.0	180	-24.0	545	
		TC164343	872	180	-24.0	180	-24.0	719	
		FG137433	750	180	-24.0	180	-24.0	490	
		FG182176	699	180	-24.0	180	-24.0	434	
		EH664871	817	170	-22.2	170	-22.2	505	
	nta-miR6151f/g/h/i	TC140583	632	324	-42.7	167	-21.7	22 6	Glucose-1-phosphate adenylyltransferase (*N. tabacum*)
		TC123361	1811	167	-21.0	167	-21.0	11	
		TC131218	818	167	-21.0	167	-21.0	19	
		TC129146	851	155	-20.9	155	-20.9	9	
miR6152	nta-miR6152a/b	TC133226	843	173	-21.3	173	-21.3	737	ATPase (*S. tuberosum*)
		TC147728	1595	173	-21.3	173	-21.3	645	
miR6153	nta-miR6153	TC141178	776	179	-25.5	179	-25.5	255	Uncharacterized protein At3g24200.2 (*A. thaliana*)
miR6154	nta-miR6154a/b	TC169226	1345	303	-44.0	155	-23.8	1213 493	Beta-D-glucan exohydrolase (*N. tabacum*)
miR6155	nta-miR6155	TC155304	729	175	-21.2	175	-21.2	210	Potassium channel (*S. lycopersicu* m)
miR6156	nta-miR6156	FS426633	460	171	-21.7	171	-21.7	100	MYB transcription factor MYB145 (*Glycine max*)
		TC128204	1441	170	-21.6	170	-21.6	929	
miR6157	nta-miR6157	TC125414	1369	171	-22.7	171	-22.7	708	Jasmonic acid-amino acid-conjugating enzyme (*N. attenuata*)
miR6158	nta-miR6158a/b/c	TC122996	1922	170	-23.3	170	-23.3	331	Biotin carboxylase (*N. tabacum*)
		FG150962	814	170	-23.3	170	-23.3	100	
miR6159	nta-miR6159	TC156785	455	306	-43.3	155	-21.7	380 194	Glycine-rich protein TomR2 (*S. lycopersicum*)
miR6160	nta-miR6160	TC122853	3924	346	-47.4	177	-24.1	3776 1477	DNA-directed RNA polymerase 1B, mitochondrial precursor (*N. ta*
miR6161	nta-miR6161a/b	TC124261	3019	325	-52.5	163	-26.6	1349 1418	ALG2-interacting protein X (*N. tabacum*)
	nta-miR6161c	TC145376	701	194	-34.2	194	-34.2	667	Avr9/Cf-9 rapidly elicited protein 76 (*N. tabacum*)
	nta-miR6161d	TC130660	873	173	-21.3	173	-21.3	576	4-diphosphocytidyl-2-C-methyl-D-erythritol kinase (*N. benthami*
		TC142702	667	173	-21.3	173	-21.3	136	
		TC167586	1556	173	-21.3	173	-21.3	809	
miR6162	nta-miR6162	TC124645	2473	164	-20.1	164	-20.1	233	Hydroxy-methylglutaryl-coenzyme A reductase (*N. tabacum*)
		TC129085	875	164	-20.1	164	-20.1	233	
		EH666128	1010	164	-20.1	164	-20.1	248	
miR6163	nta-miR6163	TC130879	989	326	-44.8	166	-23.0	214 237	DC1.2-like (*C. annuum*)
miR6164	nta-miR6164a/b	AM838512	243	187	-32.0	187	-32.0	202	Retrotransposon Tto1 DNA (*N. tabacum*)
miR827	nta-miR827	TC128315	932	170	-21.4	170	-21.4	337	CCAAT-binding transcription factor subunit B (*N. tabacum*)
		TC140030	883	170	-21.4	170	-21.4	340	
		TC143413	1544	170	-21.4	170	-21.4	185	

Table S6 Targets prediction of novel miRNAs.

miRNA name	Target gen	Gene length (n	Tot score	Tot energy	Max score	Max energ	Positions	Description
novel-miR1	TC156152	573	170	-25.7	170	-25.7	224	CC-NBS-LRR protein (*S. tuberosum*)
	TC156159	517	170	-25.7	170	-25.7	197	
	AM844322	415	170	-23.47	170	-23.47	15	
	TC166323	860	166	-25.84	166	-25.84	175	
	FS375002	510	162	-20.23	162	-20.23	189	
novel-miR2	TC141351	914	170	-24.08	170	-24.08	340	Guanine nucleotide-binding protein subunit beta-2 (*N. tabacum*)
	TC144685	849	170	-24.08	170	-24.08	179	
	TC147733	570	170	-24.08	170	-24.08	413	
novel-miR3	TC126710	953	179	-27.14	179	-27.14	835	EBP6 (*N. tabacum*)
novel-miR4	TC123427	2304	476	-63.19	162	-21.63	426 326 401	EIL5 (*Nicotiana tabacum*)
	FG137480	792	476	-63.19	162	-21.63	474 374 449	
	TC126725	954	321	-41.22	162	-20.92	495 392	
	FG163120	712	321	-41.22	162	-20.92	211 108	
novel-miR5	TC127378	1890	173	-21.12	173	-21.12	59	G6PD (*N. tabacum*)
novel-miR6	TC133182	1560	348	-62.41	190	-36.63	937 881	Transport inhibitor response 1 (*G. hirsutum*)
	TC148720	802	348	-62.41	190	-36.63	207 151	
novel-miR7	TC122895	1673	179	-25.14	179	-25.14	330	CYP (*N. tabacum*)
	TC125350	1610	179	-25.14	179	-25.14	311	
	FG640560	758	179	-25.14	179	-25.14	337	
novel-miR8	TC123057	1992	171	-20.48	171	-20.48	780	Villin 2 (*N. tabacum*)
	TC131622	1731	171	-20.48	171	-20.48	847	
novel-miR9	AM810590	480	187	-29.22	187	-29.22	151	DEAD-box ATP-dependent RNA helicase 37 (*O. sativa*)
novel-miR10	TC124312	1054	319	-42.56	162	-22.23	347 314	ASR4 (*S. lycopersicum*)
	TC146505	708	319	-42.56	162	-22.23	364 331	
	EB428262	798	319	-42.56	162	-22.23	365 332	
	TC127090	1081	793	-114.23	162	-25.08	407 440 374 308 341	DS2 protein (*S. tuberosum*)
	FS433531	603	793	-114.23	162	-25.08	462 495 429 363 396	
	TC125178	935	793	-114.23	162	-25.08	133 166 100 34 67	Abscisic stress ripening protein (*S. chacoense*)
novel-miR11	FG185054	658	170	-22.16	170	-22.16	291	Expansin-like protein precursor (*S. lycopersicum*)
novel-miR12	TC134073	1281	960	-127.53	163	-22.56	345 567 291 428 650 734	Extensin (*N. tabacum*)
	TC145834	1142	641	-84.38	163	-22.56	581 389 527 304	
	TC169113	957	478	-61.82	160	-20.61	389 527 304	
	DW002761	735	323	-43.17	163	-22.56	275 221	
novel-miR13	TC124856	980	170	-20.64	170	-20.64	415	SGT1 (*N. benthamiana*)
	TC161731	888	170	-20.64	170	-20.64	416	
	FG142982	895	170	-20.64	170	-20.64	278	
novel-miR14	TC130797	1348	867	-125.16	175	-25.58	893 992 1091 1190 1288	CDC5-like protein (*S. lycopersicum*)
	TC130688	1138	521	-74.18	175	-25.58	275 473 572	
novel-miR15	HS085450	681	338	-43.9	173	-22.74	135 97	F28C11.9 (*A. thaliana*)
novel-miR16	TC127696	1134	167	-25.04	167	-25.04	723	Phi-1 protein (*N. tabacum*)
novel-miR17	TC141992	660	175	-21.38	175	-21.38	73	Actin-related protein 3 (*N. tabacum*)
	FS430341	624	175	-21.38	175	-21.38	19	
novel-miR18	TC126568	990	165	-22.35	165	-22.35	623	IAA9 protein (*N. tabacum*)
	TC151478	878	165	-22.35	165	-22.35	668	
novel-miR19	TC126513	1278	172	-21.11	172	-21.11	519	S-adenosyl-methionine-sterol-C-methyltransferase homolog (*N. tabac*
	TC134252	805	172	-21.11	172	-21.11	607	
novel-miR20	FG155555	734	184	-24.49	184	-24.49	440	Serine/threonine protein phosphatase (*S. lycopersicum*)
	FG195049	576	176	-24.33	176	-24.33	356	
novel-miR21	FG191056	709	300	-48.06	150	-24.03	34 142	PttA (*Petunia x hybrida*)
novel-miR22	AM815929	525	174	-20.62	174	-20.62	372	WRKY transcription factor NtEIG-D48 (*N. tabacum*)
	FG193928	555	174	-20.62	174	-20.62	351	
novel-miR23	TC123450	916	159	-20.43	159	-20.43	422	Elongation factor 1-alpha (*N. tabacum*)
	TC123811	924	159	-20.43	159	-20.43	505	
	TC124472	1776	159	-20.43	159	-20.43	496	
	FG141704	883	159	-20.43	159	-20.43	415	
novel-miR24	TC128664	941	160	-26.54	160	-26.54	476	Ethylene receptor ERS homolog (*N. tabacum*)
	TC147271	538	160	-26.54	160	-26.54	487	
	TC148676	2495	160	-26.54	160	-26.54	545	
	FS427700	507	160	-26.54	160	-26.54	326	
	FS406072	613	160	-26.54	160	-26.54	460	
novel-miR25	FG162393	880	169	-21.63	169	-21.63	321	EIL1 (*Petunia x hybrida*)
	FG170485	857	169	-21.63	169	-21.63	292	
novel-miR26	TC131218	818	321	-44.87	163	-23.56	19 1	Glucose-1-phosphate adenylyltransferase (*N. tabacum*)
	TC140583	632	316	-41.8	163	-21.31	22 6	
	TC123361	1811	163	-21.31	163	-21.31	11	
	TC129146	851	155	-21.71	155	-21.71	9	
novel-miR27	TC124278	1782	175	-28.43	175	-28.43	1122	Cytochromes P450 (*N. tabacum*)
	TC122897	1727	162	-24.35	162	-24.35	1350	
	FG165245	882	153	-20.96	153	-20.96	443	
	TC122880	1806	151	-26.13	151	-26.13	1417	
	TC130596	1656	149	-20.91	149	-20.91	1397	
	TC160946	400	148	-23.23	148	-23.23	39	
novel-miR28	TC147905	2172	339	-45.52	172	-23.86	1455 1497	Extensin 1 (*N. tabacum*)
novel-miR29	TC123392	1631	174	-28.93	174	-28.93	642	Eukaryotic initiation factor (*N. tabacum*)
	TC124140	908	174	-28.93	174	-28.93	648	
	EB440487	825	174	-28.93	174	-28.93	665	
	FG190354	703	174	-28.93	174	-28.93	560	
	TC122931	1497	170	-25.89	170	-25.89	562	
novel-miR30	TC159027	844	328	-45.48	166	-25.27	804 460	Vacuolar ATP synthase 16 kDa proteolipid subunit (*N. tabacum*)
	TC124894	943	167	-22.36	167	-22.36	392	
	TC127633	955	167	-22.36	167	-22.36	406	
	TC123167	927	162	-20.21	162	-20.21	376	
	TC127603	938	162	-20.21	162	-20.21	365	
	TC138401	918	162	-20.21	162	-20.21	452	
	TC167606	877	162	-20.21	162	-20.21	308	
	FG633653	538	162	-20.21	162	-20.21	269	
	FS406852	629	162	-20.21	162	-20.21	354	

Table S7 Primers designed for stem-loop qRT-PCR analysis of miRNAs.

miRNA name	Primers
nta-miR156g	RT primer: GTCGTATCCAGTGCAGGGTCCGAGGTATTCGCACTGGATACGACAGGGAG
	Forward primer: GCGGCGGTTGGACTGAAGGGAG
	Reverse primer: CCAGTGCAGGGTCCGAGGTA
nta-miR164a	RT primer: GTCGTATCCAGTGCAGGGTCCGAGGTATTCGCACTGGATACGACTGCACG
	Forward primer: GCGGCGGTGGAGAAGCAGGGCAC
	Reverse primer: CCAGTGCAGGGTCCGAGGTA
nta-miR6149a	RT primer: GTCGTATCCAGTGCAGGGTCCGAGGTATTCGCACTGGATACGACGCCGAT
	Forward primer: GCGGCGGTTGATACGCACCTG
	Reverse primer: CCAGTGCAGGGTCCGAGGTA
nta-miR166a	RT primer: GTCGTATCCAGTGCAGGGTCCGAGGTATTCGCACTGGATACGACGGGGAA
	Forward primer: GCGGCGGTCGGACCAGGCTTC
	Reverse primer: CCAGTGCAGGGTCCGAGGTA
nta-miR169a	RT primer: GTCGTATCCAGTGCAGGGTCCGAGGTATTCGCACTGGATACGACGAGTTC
	Forward primer: GCGGCGGCTGAAGTGTTTGGGG
	Reverse primer: CCAGTGCAGGGTCCGAGGTA
novel-miR24	RT primer: GTCGTATCCAGTGCAGGGTCCGAGGTATTCGCACTGGATACGACGTGCTC
	Forward primer: GCGGCGGTCCAGCGGCTGGAAG
	Reverse primer: CCAGTGCAGGGTCCGAGGTA
5.8S rRNA	Forward primer: AGCGAAATGCGATACCT
	Reverse primer: CAACTTGCGTTCAAAGAC
